# A feed-forward loop between lncARSR and YAP activity promotes expansion of renal tumour-initiating cells

**DOI:** 10.1038/ncomms12692

**Published:** 2016-11-25

**Authors:** Le Qu, Zhenjie Wu, Yaoming Li, Zhipeng Xu, Bing Liu, Feng Liu, Yi Bao, Dengshuang Wu, Jiayi Liu, Anbang Wang, Xiaoyuan Chu, Yinghao Sun, Cheng Chen, Zhengyu Zhang, Linhui Wang

**Affiliations:** 1 Department of Urology, Jinling Hospital, Nanjing University Clinical School of Medicine, Nanjing 210002, China; 2 Department of Urology, Changzheng Hospital, Second Military Medical University, Shanghai 200003, China; 3 Department of Urology, Changhai Hospital, Second Military Medical University, Shanghai 200433, China; 4 Department of Urology, Daping Hospital, Third Military Medical University, Chongqing, China; 5 Obstetrics and Gynecology of Navy PLA General Hospital, Beijing 100048, China; 6 Department of Medical Oncology, Jinling Hospital, Nanjing University Clinical School of Medicine, Nanjing 210002, China

## Abstract

Renal tumour-initiating cells (T-ICs) contribute to tumorigenesis, progression and drug resistance of renal cell carcinoma (RCC). However, the underlying mechanism for the propagation of renal T-ICs remains unclear. Here we show that long non-coding RNA lncARSR is upregulated in primary renal T-ICs and associated with a poor prognosis of clear cell RCCs (ccRCC). Knockdown of lncARSR attenuates the self-renewal, tumorigenicity and metastasis of renal T-ICs. Conversely, forced lncARSR expression enhances T-IC properties of RCC cells. Mechanistically, the binding of lncARSR to YAP impedes LATS1-induced YAP phosphorylation and facilitates YAP nuclear translocation. Reciprocally, YAP/TEAD promotes lncARSR transcription, thus forming a feed-forward circuit. The correlation between lncARSR and YAP is validated in a ccRCC cohort, where the combination of these two parameters exhibits improved prognostic accuracy. Our findings indicate that lncARSR plays a critical role in renal T-ICs propagation and may serve as a prognostic biomarker and potential therapeutic target.

## Abstract (web summary)

Renal tumour-initiating cells (T-ICs) contribute to tumour initiation and progression. Here, the authors show that lncARSR regulates TICs by blocking LATS1-induced YAP phosphorylation facilitating YAP nuclear translocation, which promotes lncARSR transcription, thus forming a feed-forward circuit to promote TIC expansion.

## Graphical abstract

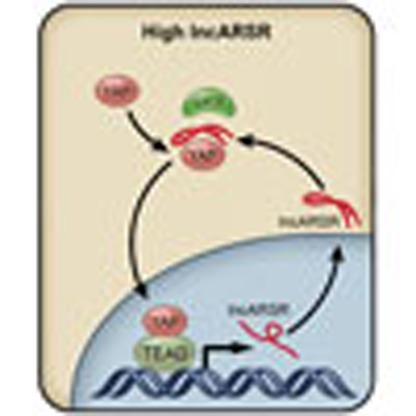

Renal cell carcinoma (RCC) is the most common kidney cancer in adults^1^ and a challenging disease with poor prognosis^2^. Increasing appreciation of cell heterogeneity within clear cell renal cell carcinoma (ccRCC)^3^ has focused attention on a distinct subpopulation of cells called tumour-initiating cells (T-ICs) or cancer stem cells (CSCs)^4^ in ccRCC. T-ICs exhibit extended self-renewal potential and tumour-initiating ability^5^. Tumours that harbour an abundant T-IC population or have high expression of stemness-related genes may signal a poor clinical outcome in RCC patients^6^,^7^. Therefore, identification of the underlying mechanisms governing renal T-ICs propagation may lead to the discovery of promising therapeutic strategies for RCC patients.

Long non-coding RNA (lncRNA) is a subgroup of transcripts with more than 200 nt and limited coding potential. lncRNAs modulate biological process via diverse mechanisms^8^, including mobilizing transcriptional co-regulators or chromatin-modifying complex^9^,^10^ at transcription level, and interacting with RNAs^11^,^12^,^13^ and protein complex^14^,^15^ or modifying signal proteins^16^,^17^ at post-transcription level. Several lncRNAs have been reported to regulate the self-renewal of T-ICs especially liver T-ICs^18^,^19^,^20^. Nevertheless, the role of lncRNA in the regulation of renal T-ICs remains unknown.

lncARSR (lncRNA Activated in RCC with Sunitinib Resistance, *ENST00000424980*) was a newly identified lncRNA to promote the sunitinib resistance of RCC in our previous study^21^. Accumulating evidence indicated that T-ICs surviving from drug therapy and giving rise to tumour regrowth might be a major culprit for therapeutic resistance^22^,^23^,^24^,^25^. Indeed, the expression signature of stem cell^26^,^27^,^28^ or targets of Nanog, Oct4, Sox2 and c-Myc (NOSM) in human ESCs^29^,^30^,^31^ were significantly enriched in our mRNA profile of sunitinib-resistant RCC cells (GSE69535) (Supplementary Fig. 1a), prompting us to explore the role of lncARSR in renal T-ICs.

In this study, we first find that lncARSR is highly expressed in primary renal T-ICs and predicts poor prognosis. Next, by using loss-of-function analysis in T-ICs and gain-of-function analysis in RCC cells, we demonstrate that lncARSR promotes the self-renewal capacity, tumorigenicity and metastasis of renal T-ICs. Further mechanism study reveals that lncARSR interacts with Yes-associated protein (YAP) to block its phosphorylation by LATS1, facilitating YAP nuclear translocation. Interestingly, we find that YAP in turn promotes the transcription of lncARSR, forming a feed-forward loop. Clinical investigation also confirms the correlation between lncARSR and YAP, and demonstrates the value of combining lncARSR and YAP to improve the prognostic accuracy for RCC patients. Altogether, we discover that lncARSR promotes the expansion of renal T-ICs via interacting with YAP.

## Results

### lncARSR is upregulated in T-ICs and predicts poor prognosis

**Figure 1:**
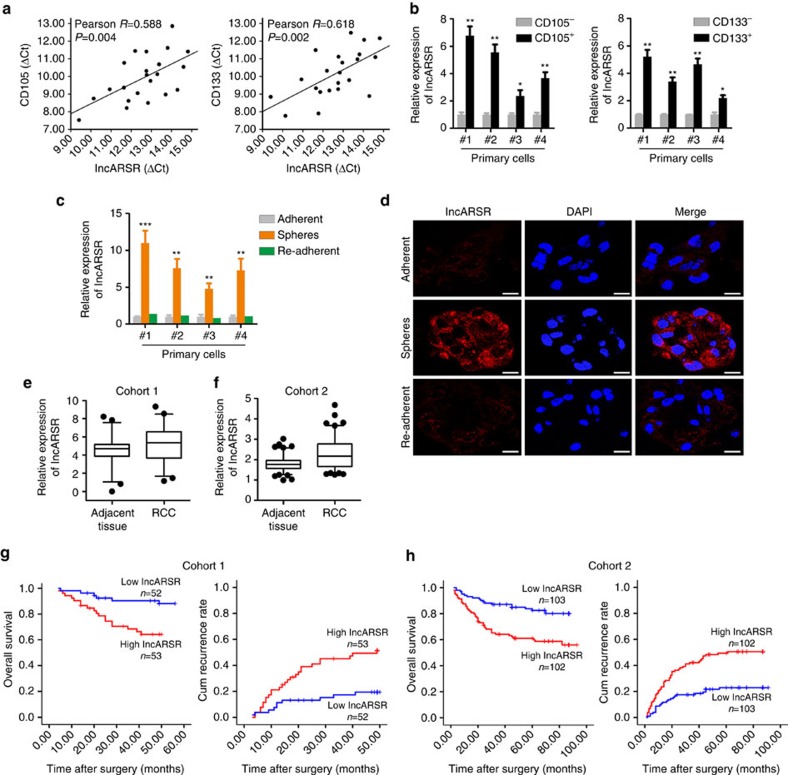
lncARSR is upregulated in T-ICs and predicts poor prognosis. (**a**) The correlation between the transcription level of lncARSR and CD105 (left) or CD133 (right) in primary ccRCC cells (*n*=22) was determined by qRT–PCR analysis. Data were normalized to β-actin as ΔCt and analysed by Spearman's correlation analysis. (**b**) qRT–PCR analysis of lncARSR in MACS sorted CD105^+^ (left) or CD133^+^ (right) primary ccRCC cells relative to negative cells. Data are represented as mean±s.d.; *P<0.05 and ***P<0.001; two-tailed Student's *t*-test. (**c**) qRT–PCR analysis of lncARSR in primary ccRCC adherent, spheres and re-adherent cells. Data are represented as mean±s.d.; **P<0.01 and ***P<0.001; two-tailed Student's *t*-test. (**d**) FISH analysis of lncARSR in primary ccRCC #4 adherent and sphere cells using biotin-labelled LNA probe. The nuclei were stained with DAPI. Scale bar, 20 μm. (**e**) lncARSR expression in human ccRCC tissues and adjacent tissues determined by qRT–PCR analysis in cohort 1 (*n*=105, *P*<0.001, Mann–Whitney *U*-test). The lncARSR expression was normalized to β-actin (ΔCt) and compared with the maximum ΔCt. Data are presented as −ΔΔCt. The horizontal lines in the box plots represent the median, the boxes represent the interquartile range and the whiskers represent the 2.5th and 97.5th percentiles. (**f**) lncARSR expression in human ccRCC tissues and adjacent tissues determined by RNA in situ hybridization (ISH) analysis in cohort 2 (*n*=205, *P*<0.001, Mann–Whitney *U*-test). The lncARSR expression was normalized to the minimum signal of lncARSR positive staining. The horizontal lines in the box plots represent the median, the boxes represent the interquartile range, and the whiskers represent the 2.5th and 97.5th percentiles. (**g**) Kaplan–Meier analysis of overall survival (left, *P*<0.001, log-rank test) or recurrence rate (right, *P*<0.001, log-rank test) of ccRCC patients in low and high lncARSR groups in cohort 1. (**h**) Kaplan–Meier analysis of overall survival (left, *P*<0.001, log-rank test) or recurrence rate (right, *P*<0.001, log-rank test) of ccRCC patients in low and high lncARSR groups in cohort 2.

CD105 and CD133 are well-accepted renal T-IC markers^32^. In tumour cells isolated from primary ccRCC tissues, pearson correlation analysis revealed that lncARSR levels were positively correlated with the expression of CD105 and CD133 (Fig. 1a). To determine the expression of lncARSR in renal T-ICs, we enriched T-ICs by flow cytometry sorting or sphere formation (Supplementary Fig. 1b,c). As shown in Fig. 1b, lncARSR levels were upregulated in sorted CD105^+^ or CD133^+^ primary ccRCC cells. Compared with adherent cells, lncARSR expression was increased in RCC spheres derived from human primary ccRCC cells. Notably, lncARSR level was reduced to origin level when the spheres were reattached (Fig. 1c,d). Several RCC cell lines showed the similar results (Supplementary Fig. 1d,e). These data indicated that lncARSR was preferentially upregulated in renal T-ICs.

To investigate the clinical significance of lncARSR, we determined lncARSR expression in a total of 310 ccRCC tissues from two independent cohorts. The average level of lncARSR was higher in ccRCC tumours than adjacent non-tumour tissues determined by quantitative PCR with reverse transcription (qRT–PCR) and *in situ* hybridization (ISH) (Fig. 1e,f; Supplementary Fig. 1f and Supplementary Table 1). Notably, lncARSR expression was elevated in poorly differentiated ccRCC tumours compared with well-differentiated tumours (Supplementary Fig. 1g), prompting a putative role of lncARSR in renal T-ICs. Correlation regression analysis revealed that high lncARSR expression in ccRCC tissues was associated with aggressive clinical features (Supplementary Tables 2 and 3). Moreover, patients with higher lncARSR levels exhibited worse overall survival and shorter time to recurrence (Fig. 1g,h). Multivariate analysis manifested that high lncARSR level was an independent predictor for poor prognosis of ccRCC patients (Supplementary Tables 4–7).

### lncARSR is required for the maintenance of renal T-ICs

**Figure 2:**
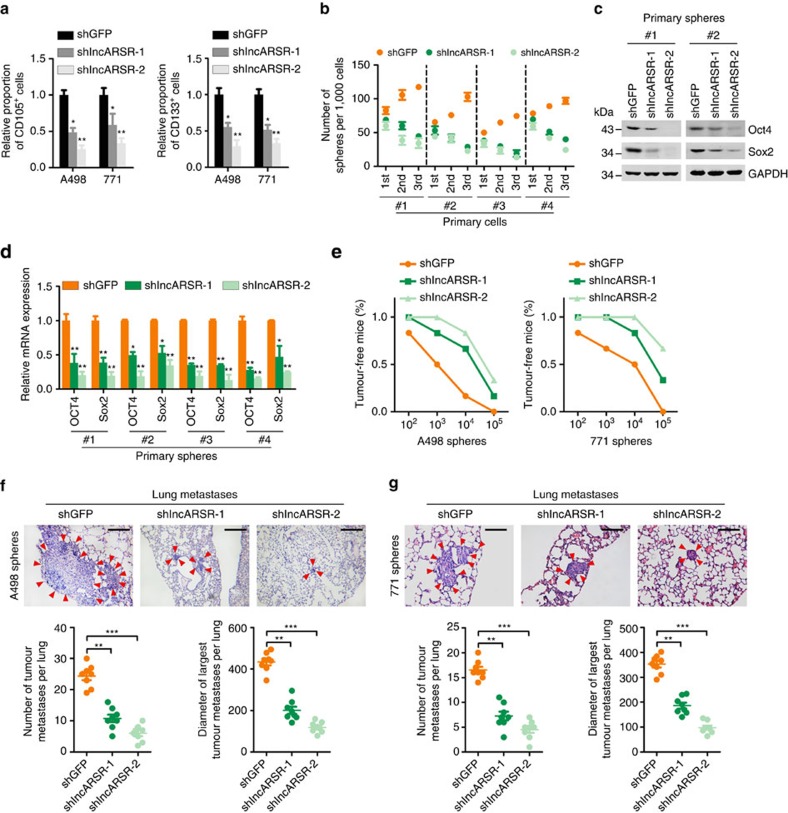
lncARSR is required for the maintenance of renal T-ICs. (**a**) Flow cytometric analysis of the proportion of CD105^+^ (left) or CD133^+^ (right) cells in lncARSR-knockdown and control RCC cells (*n*=3). Data are represented as mean±s.d.; *P<0.05 and **P<0.01; two-tailed Student's *t*-test. (**b**) Spheres formation assay of lncARSR-knockdown and control primary ccRCC cells (*n*=3). The number of primary, secondary and tertiary passaged spheres was counted after 7 days. (**c**) Western blot analysis of Oct4 and Sox2 in lncARSR-knockdown and control primary ccRCC spheres. GAPDH acted as a loading control. (**d**) qRT–PCR analysis of indicated mRNAs in lncARSR knockdown and control primary ccRCC spheres. Data are represented as mean±s.d.; *P<0.05 and **P<0.01; two-tailed Student's *t*-test. (**e**) *In vivo* limiting dilution assay of lncARSR knockdown and control sphere-derived RCC cells. Tumours were observed over 2 months; *n*=6 for each group. (**f**,**g**) Representative microscopic images of pulmonary metastatic lesions at 12 weeks after the injection of indicated sphere-derived RCC cells into the tail vein of nude mice (upper). Red arrows indicate lung metastatic tumours. Scale bar, 200 μm. The number (lower left) and diameter (lower right) of lung metastatic tumours in each group (*n*=8) were calculated. Data are represented as mean±s.d.; **P<0.01 and ***P<0.001; two-tailed Student's *t*-test.

To explore the potential role of lncARSR in renal T-ICs, we suppressed lncARSR expression utilizing two independent lentivirus-based short hairpin RNAs (shRNAs) in primary ccRCC cells and cell lines (Supplementary Fig. 2a). Flow cytometry analysis showed that knockdown of lncARSR decreased the proportion of CD105^+^ or CD133^+^ cells (Fig. 2a). Primary ccRCC spheres with lncARSR knockdown exhibited impaired self-renewal capacity on serial passage and decreased expression of pluripotent transcription factors (Fig. 2b–d). Similar results were also observed in RCC cell lines (Supplementary Fig. 2b,c), indicating that knockdown of lncARSR attenuated the self-renewal capacity of renal T-ICs.

To further determine the effect of lncARSR on the tumorigenicity of renal T-ICs, sphere-derived shlncARSR or shGFP cells were inoculated into nude mice. *In vivo* limiting dilution assay revealed that suppression of lncARSR significantly reduced tumour incidence and T-IC frequency (Fig. 2e and Supplementary Table 8) consistent with the cell culture studies. Moreover, RCC cells derived from the shlncARSR-xenografts showed impaired ability to form secondary tumours by serial passage compared to control xenografts (tumour incidence: shGFP, 4/4; shlncARSR-1, 0/4; shlncARSR-2, 0/4) (Supplementary Fig. 2d), indicating that interference of lncARSR impaired the tumour formation ability of renal T-ICs. Furthermore, cells from dissociated shlncARSR spheres formed decreased number and size of pulmonary metastatic lesions in mice (Fig. 2f,g). Collectively, these results demonstrated that lncARSR played a critical role in the maintenance of renal T-ICs.

### lncARSR promotes renal T-ICs expansion

**Figure 3:**
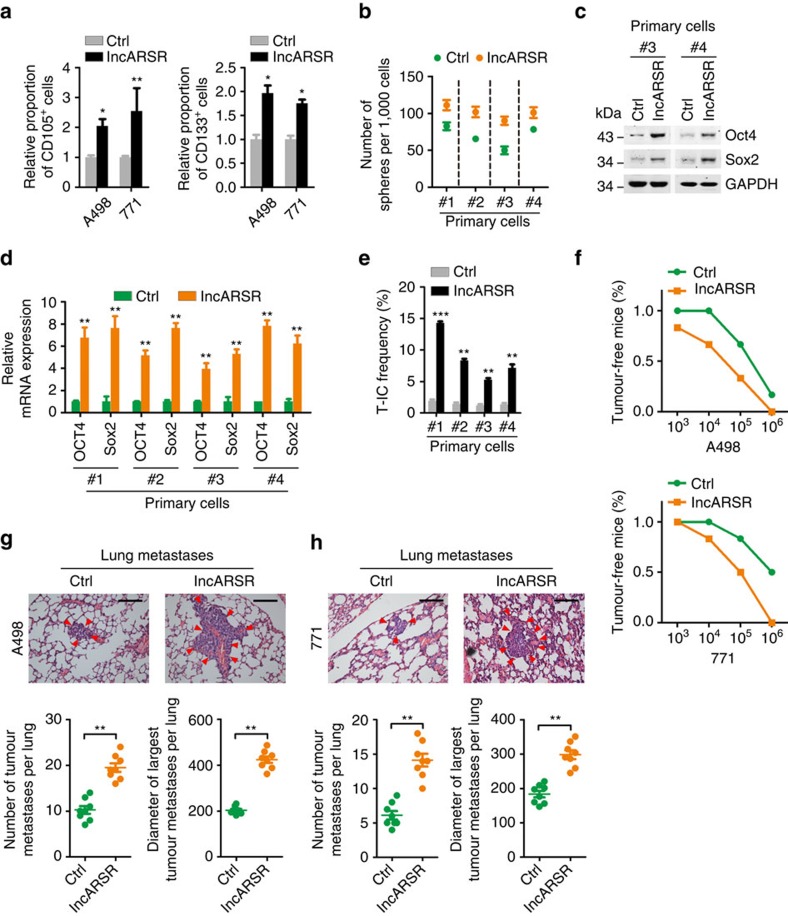
lncARSR promotes renal T-ICs expansion. (**a**) Flow cytometric analysis of the proportion of CD105^+^ (left) or CD133^+^ (right) cells in lncARSR-overexpressing and control RCC cells (*n*=3). Data are represented as mean±s.d.; *P<0.05 and **P<0.01; two-tailed Student's *t*-test. (**b**) Spheres formation assay of lncARSR-overexpressing and control primary ccRCC cells (*n*=3). The number of spheres was counted after 7 days. (**c**) Western blot analysis of Oct4 and Sox2 in lncARSR-overexpressing and control primary ccRCC cells. (**d**) qRT–PCR analysis of indicated mRNAs in lncARSR-overexpressing and control primary ccRCC cells. Data are represented as mean±s.d.; **P<0.01; two-tailed Student's *t*-test. (**e**) *In vitro* limiting dilution assay of lncARSR-overexpressing and control primary ccRCC cells. The results were shown as natural logarithm of the proportion of T-ICs. (**f**) *In vivo* limiting dilution assay of lncARSR-overexpressing and control RCC cells. Tumours were observed over 2 months; *n*=6 for each group. (**g**,**h**) Representative microscopic images of pulmonary metastatic lesions at 12 weeks after the injection of indicated RCC cells into the tail vein of nude mice (upper). Red arrows indicate lung metastatic tumours (left). Scale bar, 200 μm. The number (lower left) and diameter (lower right) of lung metastatic tumours in each group (*n*=8) were calculated. Data are represented as mean±s.d.; **P<0.01; two-tailed Student's *t*-test.

Next, we overexpressed lncARSR in RCC cells by lentivirus delivery (Supplementary Fig. 3a). lncARSR-overexpressing cells harboured expanded proportion of CD105^+^ or CD133^+^ cells (Fig. 3a). Primary ccRCC cells with lncARSR overexpression exhibited enhanced sphere-forming ability and increased expression of pluripotent transcription factors (Fig. 3b–d). *In vitro* limiting dilution assay revealed that lncARSR overexpression increased the T-IC frequency of primary ccRCC cells (Fig. 3e). Similar results were also achieved in RCC cell lines (Supplementary Fig. 3b–d). Moreover, lncARSR overexpression yielded an enhanced tumorigenicity and pulmonary metastasis of RCC cells *in vivo* (Fig. 3f–h and Supplementary Table 9). These data indicated that lncARSR facilitates the expansion of renal T-ICs.

### lncARSR physically interacts with YAP

Our previous data demonstrated that lncARSR was mainly localized in cytoplasm and could act as competing endogenous RNA to sequestrate miRNA^21^. However, knockdown of Dicer had little effect on lncARSR-induced upregulation of stemness genes (Supplementary Fig. 4a,b), excluding the role of miRNA in lncARSR-induced T-IC property.

**Figure 4:**
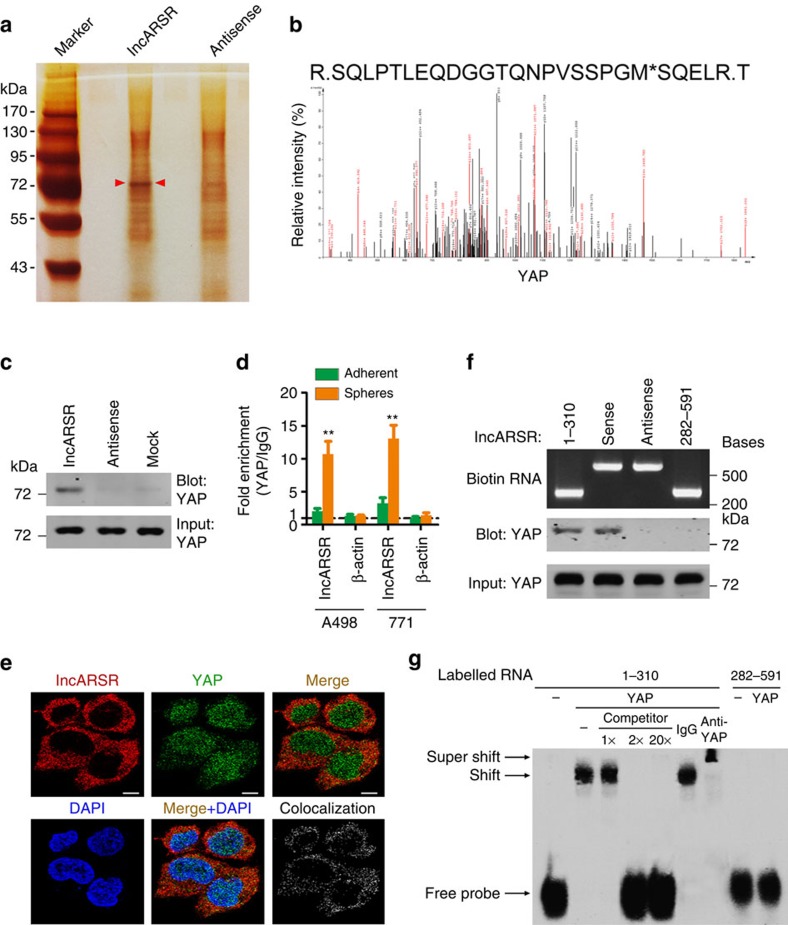
lncARSR physically interacts with YAP. (**a**) RNA pull-down assays were performed with cytoplasmic lysates of RCC spheres using full-length lncARSR and antisense RNA probes, followed by mass spectrum. Red arrows indicate target band. (**b**) MS/MS profiles of target band (corresponding peptide sequences of YAP) retrieved by lncARSR. (**c**) Western blot analysis of YAP in RNA pull-down precipitates retrieved by biotin-labelled lncARSR or antisense RNA from the cytoplasmic lysates of RCC spheres. (**d**) RIP assay of the enrichment of YAP on lncARSR relative to IgG in the cytoplasmic lysates of adherent and spheres RCC cells (*n*=3). β-action acted as a loading control. Data are represented as mean±s.d.; **P<0.01; two-tailed Student's *t*-test. (**e**) RNA FISH analysis of lncARSR (red) and immunofluorescence detection of YAP (green) in A498 spheres. The rightmost graph shows the co-localized signals between the red signal (lncARSR) and the green signal (YAP). Pearson's correlation: *R*=0.658382. Scale bar, 10 μm. (**f**) Upper: gel electrophoresis of *in vitro* transcribed biotin-labelled RNA of full-length and truncated lncARSR. Lower: western blot analysis of YAP in RNA pull-down precipitates retrieved by different fragments of lncARSR probe. (**g**) RNA EMSA assays of the interaction of different fragments of lncARSR and YAP. Biotin-labelled RNA containing nucleotides 1–310 or 282–591 bp of lncARSR acted as probes. Non-labelled RNA probes acted as competitors. Anti-YAP antibody acted for super-shift experiment. ‘Shift' indicates the shift in mobility induced by the biotin-labelled RNA probe and YAP complex. ‘Super-shift' indicates the super-shift in mobility induced by the biotin-labelled RNA probe, YAP and anti-YAP antibody complex.

To dissect the mechanism underlying the promotive role of lncARSR in renal T-IC, we performed RNA pull-down assay to seek lncARSR-interacting proteins (Fig. 4a). The band specifically pulled down by lncARSR was subject to mass spectrometry analysis (Supplementary Data 1). Nine proteins that localized in cytoplasm with corresponding molecular mass were selected and subjected to loss-of-function analysis (Supplementary Fig. 4c). Four out of the nine candidate proteins were required for the sphere-forming ability of RCC cells (Supplementary Fig. 4d), but only YAP was further reproducibly detected by independent RNA pull-down assays (Fig. 4b,c and Supplementary Fig. 4e). RNA immunoprecipitation (RIP) assay confirmed the interaction between YAP and lncARSR in RCC spheres (Fig. 4d). Consistently, lncARSR co-localized with YAP in the cytoplasm by RNA fluorescence *in situ* hybridization (RNA FISH) and immunofluorescence (Fig. 4e), which was validated by quantitative co-localization analysis (Pearson's correlation *R*=0.658382)^33^. Furthermore, RNA pull-down assay with truncated lncARSR mutants demonstrated that the 5′ segment of lncARSR (nucleotides 1–310) was responsible for its interaction with YAP (Fig. 4f). RNA electrophoretic mobility shift assay (EMSA) revealed that RNA–YAP complex was efficiently competed by unlabelled lncARSR probes (nucleotides 1–310) and super-shifted by anti-YAP antibody (Fig. 4g), further confirming the binding of the 5′ segment of lncARSR to YAP. Moreover, data from EMSA suggested a 1:1 stoichiometry between the lncARSR and YAP. RNA folding analysis^34^ of lncARSR 5′-segment predicted a stable stem-loop structure (Supplementary Fig. 4f), which might provide the spatial conformations for its binding with YAP.

### YAP is responsible for lncARSR-mediated T-IC properties

**Figure 5:**
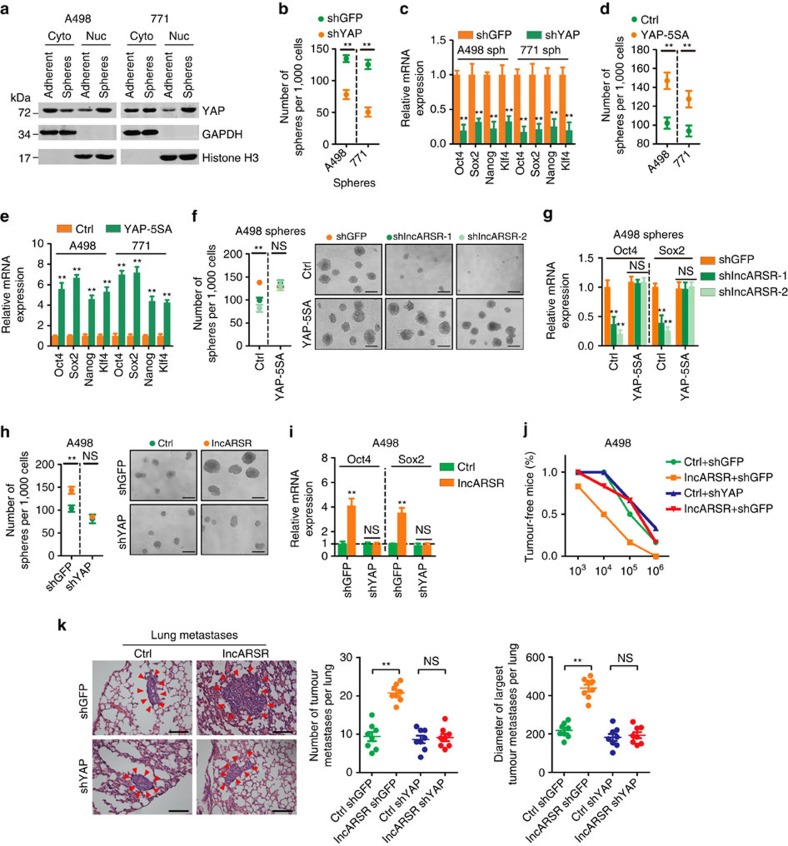
YAP is responsible for lncARSR-mediated T-IC properties. (**a**) Western blot analysis of YAP in subcellular fractions of RCC adherent and sphere cells. GAPDH and Histone H3 acted as cytoplasm and nucleus marker, respectively. (**b**) Spheres formation assay of YAP-knockdown and control RCC spheres (*n*=3). The number of spheres was counted after 7 days. (**c**) qRT–PCR analysis of indicated mRNAs in YAP-knockdown and control RCC spheres (*n*=3). (**d**) Spheres formation assay of YAP-5SA-overexpressing and control RCC cells (*n*=3). The number of spheres was counted after 7 days. (**e**) qRT–PCR analysis of indicated mRNAs in YAP-5SA-overexpressing and control RCC cells (*n*=3). (**f**) Spheres formation assay of A498 spheres transfected with indicated plasmids (*n*=3). The number of spheres was counted after 7 days (left). Representative images of spheres are shown (right). Scale bar, 200 μm. (**g**) qRT–PCR analysis of Oct4 and Sox2 in A498 spheres transfected with indicated plasmids after 48 h (*n*=3). (**h**) Spheres formation assay of A498 cells transfected with indicated plasmids (*n*=3). The number of spheres was counted after 7 days (left). Representative images of spheres are shown (right). Scale bar, 200 μm. (**i**) qRT–PCR analysis of Oct4 and Sox2 in A498 cells transfected with indicated plasmids after 48 h (*n*=3). (**b**–**i**) Data are represented as mean±s.d.; **P<0.01; two-tailed Student's *t*-test. (**j**) *In vivo* limiting dilution assay of indicated RCC cells. Tumours were observed over 2 months; *n*=6 for each group. (**k**) Representative microscopic images of pulmonary metastatic lesions at 12 weeks after the injection of indicated RCC cells into the tail vein of nude mice (upper). Red arrows indicate lung metastatic tumours (left). Scale bar, 200 μm. The number (middle) and diameter (right) of lung metastatic tumours in each group (*n*=8) were calculated. Data are represented as mean±s.d.; **P<0.01; two-tailed Student's *t*-test.

YAP, a transcription co-activator in Hippo signalling^35^,^36^, has been reported to play critical roles in T-ICs expansion in various cancers^37^,^38^. Indeed, the expression of YAP was nuclear-enriched in renal T-ICs (Fig. 5a). Knockdown of YAP impaired the proportion of CD105^+^ or CD133^+^ cells and attenuated the sphere-forming capacity and the expression of pluripotent transcription factors in RCC spheres (Fig. 5b,c and Supplementary Fig. 5a,b). Conversely, introduction of constitutively activated YAP (YAP-5SA)^39^ increased the T-IC properties of RCC cells (Fig. 5d,e and Supplementary Fig. 5c,d), indicating a critical role of YAP in the maintenance and expansion of renal T-ICs.

Notably, introduction of YAP-5SA restored the self-renewal capacity and the expression of pluripotent transcription factors in lncARSR-knockdown RCC spheres (Fig. 5f,g). While knockdown of YAP eliminated the discrepant T-IC properties triggered by lncARSR overexpression (Fig. 5h,i) or depletion (Supplementary Fig. 5e,f), indicating that YAP is required for lncARSR-mediated renal T-IC maintenance and expansion. More importantly, YAP depletion abrogated the lncARSR-enhanced tumorigenicity and pulmonary metastasis of RCC cells *in vivo* (Fig. 5j,k and Supplementary Table 10). Together, these data demonstrate that YAP is responsible for lncARSR-promoted renal T-IC expansion.

### lncARSR blocks LATS1-mediated YAP phosphorylation

**Figure 6:**
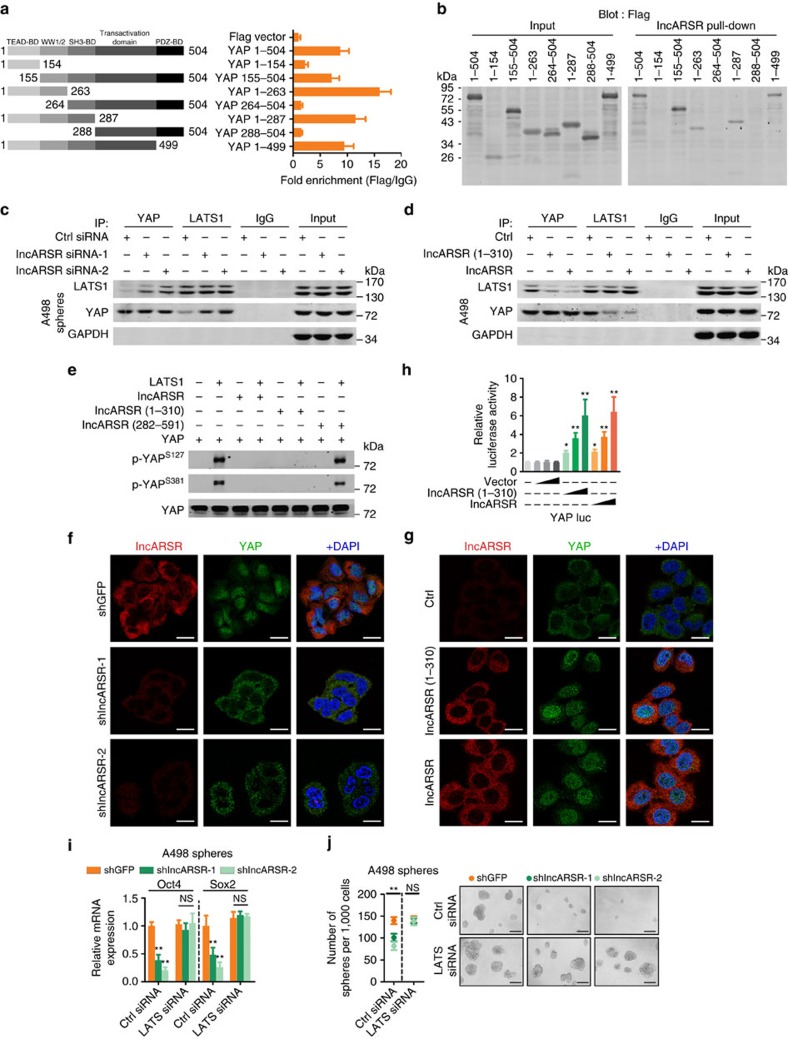
lncARSR blocks LATS1-mediated YAP phosphorylation. (**a**) Left: schematic diagram of different fragments of YAP was shown. Right: RIP assay of the enrichment of different YAP fragments on lncARSR relative to IgG in A498 spheres transfected with different fragments of YAP plasmids (*n*=3). BD, binding domain. (**b**) Western blot analysis of Flag in whole lysates of A498 spheres (input, left) and RNA pull-down precipitates retrieved by biotin-labelled lncARSR from A498 spheres transfected with different fragments of YAP-Flag plasmids. (**c**) Coimmunoprecipitation of YAP and LATS1 in lysates of lncARSR-knockdown and control A498 spheres. GAPDH acted as a loading control. (**d**) Coimmunoprecipitation of YAP and LATS1 in lysates of A498 cells with lncARSR overexpression or 5′ segment (nucleotides 1–310) overexpression. GAPDH acted as a loading control. (**e**) *In vitro* phosphorylation assay with purified active LATS1 showing the effects of different fragments of lncARSR on LATS1-induced YAP phosphorylation. (**f**) RNA FISH analysis of lncARSR and immunofluorescence detection of YAP in lncARSR-knockdown and control A498 spheres. Scale bar, 20 μm. (**g**) RNA FISH analysis of lncARSR and immunofluorescence detection of YAP in A498 cells with lncARSR or 5′ segment (nucleotides 1–310) overexpression. Scale bar, 20 μm. (**h**) A498 cells were transfected with YAP/TAZ luciferase reporter plasmid (YAP/TAZ luc), together with increasing concentrations of indicated plasmids, and subjected to luciferase reporter assay (*n*=3). Data were normalized against Renilla luciferase activity and represented as mean±s.d.; *P<0.05 and **P<0.01; two-tailed Student's *t*-test. (**i**) qRT–PCR analysis of Oct4 and Sox2 in A498 spheres transfected with indicated siRNA and plasmid after 48 h (*n*=3). (**j**) Spheres formation assay of A498 spheres transfected with indicated siRNA and plasmid (*n*=3). The number of spheres was counted after 7 days (left). Representative images of spheres are shown (right). Scale bar, 200 μm. (**i**,**j**) Data are represented as mean±s.d.; **P<0.01; two-tailed Student's *t*-test.

To determine how lncARSR regulated YAP activity, we constructed truncated YAP mutants to unravel its binding sites with lncARSR. RIP assay revealed that the WW1/2 domain of YAP (residues 155–263) was required for its interaction with lncARSR (Fig. 6a), which was confirmed by RNA pull-down assay (Fig. 6b). The WW1/2 domain of YAP has been reported to interact with LATS1 (large tumour suppressor kinase 1), which could induce YAP phosphorylation and subsequent cytoplasmic retention^40^. Therefore, we speculated that lncARSR might block LATS1–YAP interaction to promote YAP nuclear translocation. Immunoprecipitation assay showed that lncARSR knockdown facilitated the interaction between LATS1 and YAP in RCC spheres (Fig. 6c and Supplementary Fig. 6a,b), and overexpression of lncARSR or its 5′-segment attenuated LATS1–YAP interaction in adherent cells (Fig. 6d and Supplementary Fig. 6c). As a result, YAP phosphorylation was increased by lncARSR knockdown and suppressed on overexpression of lncARSR or its 5′-segment (Supplementary Fig. 6d,e). Furthermore, *in vitro* kinase assay showed that lncARSR or its 5′-segment could protect YAP from phosphorylation by purified active LATS1 (Fig. 6e). As expected, YAP nuclear translocation was enhanced on lncARSR overexpression and attenuated upon lncARSR knockdown (Fig. 6f,g; Supplementary Fig. 6f,g). YAP translocates into the nucleus mainly serving as a transcription co-activator. As shown in Fig. 6h and Supplementary Fig. 6h, overexpression of lncARSR or its 5′-segment enhanced YAP luciferase reporter^41^ activity and the expression of YAP downstream genes. Notably, knockdown of LATS restored the self-renewal capacity and the expression of pluripotent transcription factors in lncARSR-knockdown spheres (Fig. 6i,j and Supplementary Fig. 6i). Together, these results demonstrated that lncARSR–YAP interaction prevented the phosphorylation of YAP by LATS1 and facilitated YAP nuclear translocation to promote renal T-IC properties.

### YAP/TEAD complex transactivates lncARSR

**Figure 7:**
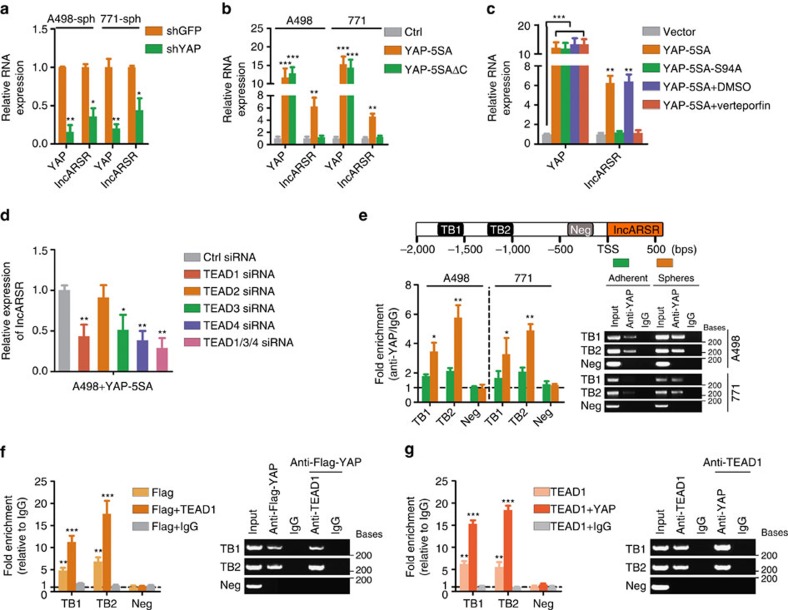
YAP/TEAD complex transactivates lncARSR. (**a**) qRT–PCR analysis of YAP and lncARSR in RCC spheres transfected with shYAP or shGFP plasmid after 48 h (*n*=3). (**b**) qRT–PCR analysis of YAP and lncARSR in RCC cells transfected with YAP-5SA plasmid or YAP-5SAΔC plasmid after 48 h (*n*=3). (**c**) qRT–PCR analysis of YAP and lncARSR in A498 cells transfected with indicated plasmids or treated with Verteporfin (1 μM) for 48 h (*n*=3). (**d**) qRT–PCR analysis of lncARSR in YAP-5SA-overexpressing A498 cells transfected with indicated siRNA after 48 h (*n*=3). (**e**) Upper: putative TEAD-binding sites on the promoter region of lncARSR. Lower: ChIP assay of the enrichment of YAP on lncARSR promoter relative to IgG in RCC adherent and sphere cells (*n*=3). A random region without TB sites acted as a negative control (Neg). (**f**) ChIP–re-ChIP assay was performed using anti-Flag-YAP antibody first (Flag-YAP). The eluants were then subjected to a second ChIP assay using anti-TEAD1 antibody (Flag-YAP+TEAD1) or control IgG antibody (Flag-YAP+IgG) (*n*=3). (**g**) ChIP-re-ChIP assay was performed using anti-TEAD1 antibody first (TEAD1). The eluants were then subjected to a second ChIP assay using anti-YAP antibody (TEAD1+YAP) or control IgG antibody (TEAD1+IgG) (*n*=3). (**a**–**g**) Data are represented as mean±s.d.; *P<0.05, **P<0.01 and ***P<0.001; two-tailed Student's *t*-test.

Regulatory feed-forward loops have been reported to involve in tumour initiation and progression^42^,^43^. As shown in Fig. 7a, lncARSR levels were repressed on YAP deletion in RCC spheres. While lncARSR levels were enhanced by introducing YAP-5SA but not YAP-5SAΔC^39^, a YAP mutant lacking transactivation domain (Fig. 7b and Supplementary Fig. 7a), indicating that YAP might modulate lncARSR transcription. It has been reported that YAP transactivates target genes mainly through interacting with TEA domain family (TEAD)^44^. Intriguingly, overexpression of YAP-5SA-S94A (mutant without TEAD-binding capacity)^44^ or Verteporfin treatment^45^, a drug selectively disrupts YAP-TEAD binding, failed to promote lncARSR transcription (Fig. 7c). Interference of TEAD family members revealed that TEAD1, TEAD3 and TEAD4 but not TEAD2 were required for YAP-induced lncARSR expression and the upregulation of lncARSR in RCC spheres (Fig. 7d and Supplementary Fig. 7b,c). Notably, bioinformatics analysis predicted two conserved TEAD-binding (TB) sites in lncARSR promoter region (Fig. 7e, upper panel). Chromatin immunoprecipitation (ChIP) assay demonstrated that YAP was enriched on the predicted TB sites in lncARSR promoter, and the enrichment was significantly upregulated in spheres (Fig. 7e, lower panel). Furthermore, ChIP–re-ChIP assays confirmed the co-occupancy of YAP and TEAD1 on lncARSR promoter (Fig. 7f,g). Collectively, these data indicated that YAP/TEAD complex bound to lncARSR promoter and promoted lncARSR transcription, therefore forming a positive feedback circuit in renal T-ICs.

### Combining lncARSR and YAP exhibits improved prognostic value

**Figure 8:**
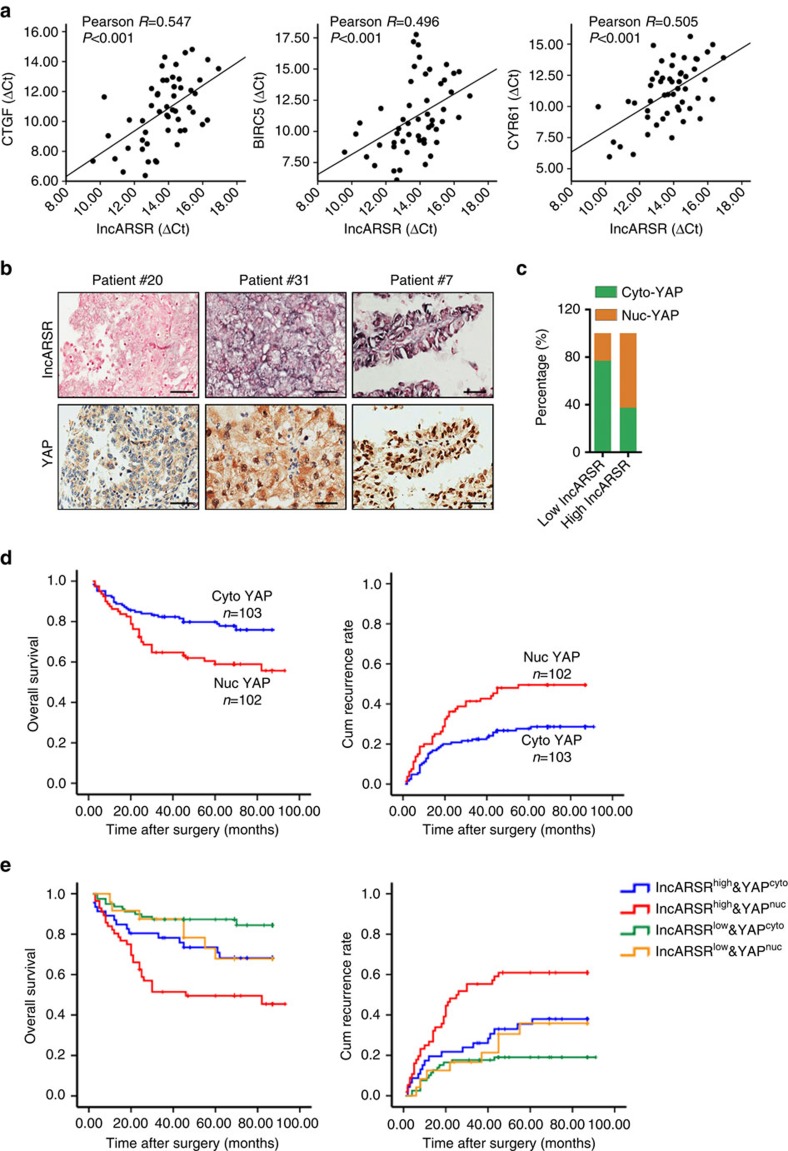
Combining lncARSR and YAP exhibits improved prognostic value. (**a**) The correlation between the transcription level of lncARSR and CTGF (left), BIRC5 (middle) and CYR61 (right) in human ccRCC tissues (*n*=52) was determined by qRT–PCR analysis. Data were normalized to β-actin as ΔCt and analysed by Spearman's correlation analysis. (**b**) Representative images of lncARSR ISH and YAP immunohistochemistry using consecutive sections of human ccRCC tissues. Scale bar, 50 μm. (**c**) Relative percentages of nuclear and cytoplasmic YAP in high (62.7%) and low (23.3%) lncARSR groups in cohort 2 (*P*<0.001). (**d**) Kaplan–Meier analysis of overall survival (left, *P*=0.005, log-rank test) or recurrence rate (right, *P*=0.002, log-rank test) of ccRCC patients with cytoplasmic or nuclear YAP in cohort 2. (**e**) Kaplan–Meier analysis of overall survival (left, lncARSR^high^YAP^high^ versus lncARSR^high^YAP^low^, *P*=0.039, log-rank test; lncARSR^low^YAP^high^ versus lncARSR^low^YAP^low^, *P*=0.093, log-rank test) or recurrence rate (right, lncARSR^high^YAP^high^ versus lncARSR^high^YAP^low^, *P*=0.014, log-rank test; lncARSR^low^YAP^high^ versus lncARSR^low^YAP^low^, *P*=0.173, log-rank test) of ccRCC patients with high or low lncARSR and cytoplasmic or nuclear YAP.

To verify the correlation between lncARSR and YAP activity in clinical samples, we examined the RNA levels of lncARSR and YAP target genes in 52 human ccRCC tumour specimens. As shown in Fig. 8a, the levels of lncARSR were positively correlated with the levels of YAP target genes CTGF, BIRC5 and CYR61. Moreover, we performed immunochemistry staining of YAP in ccRCC tissue microarray, which had been subjected to ISH analysis of lncARSR. Expression of lncARSR was positively correlated with nuclear accumulation of YAP in ccRCC tissues (Fig. 8b and Supplementary Table 11 and Supplementary Fig. 8a). The ratio of patients with nuclear YAP enrichment was increased in high lncARSR group compared with that in low lncARSR group (Fig. 8c). Although either high lncARSR (Fig. 1h) or nuclear YAP in ccRCC predicts a poor prognosis (Fig. 8d), ccRCC patients with both elevated lncARSR level and nuclear YAP expression displayed even worse prognosis (Fig. 8e), indicating a better prognostic value of combining the two parameters in comparison with lncARSR or YAP alone.

**Figure 9:**
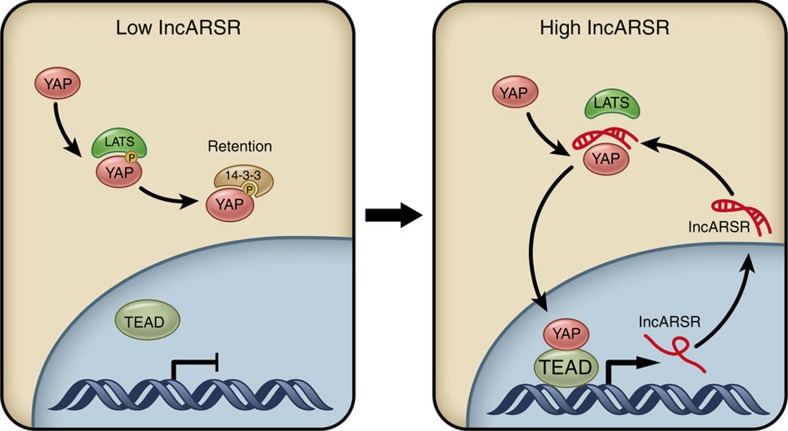
Schematic diagram of lncARSR–YAP signaling circuit in renal T-ICs. lncARSR overexpression impedes LATS-YAP interaction to facilitate the nuclear translocation of YAP, which in turn transactivates lncARSR expression, forming a feed-forward loop to promote the expansion of renal T-ICs.

In aggregate, our results unravelled that lncARSR-promoted renal T-ICs propagation via impeding LATS1–YAP interaction to facilitate YAP nuclear translocation, which reciprocally enhanced lncARSR transcription, thus forming a feed-forward circuit in renal T-ICs (Fig. 9).

## Discussion

Most cancer therapies fail to eradicate tumours due to the existence of T-ICs. However, the understanding of regulatory mechanisms for T-ICs is limited. In this study, we elaborated the critical role of lncARSR in renal T-ICs and the underlying mechanism. We also demonstrated the value of combining lncARSR and YAP to improve the prognostic accuracy for RCC patients. To our knowledge, this is the first report for lncRNA in the regulation of renal T-ICs.

Accumulating evidence shows that lncRNAs could serve as molecular scaffolds to facilitate multiple proteins interaction, such as lncRNA-NEAT1, which is an essential molecular scaffold for the formation of paraspeckles^46^, but lncRNA *per se* have rarely been reported to exhibit direct regulatory effect. In this study, we found that lncARSR, unlike scaffold lncRNAs, bound specifically to the WW1/2 domain of YAP, thus protecting YAP from interaction and phosphorylation by LATS1. Our data characterized lncARSR as a direct signal transducer through acting on the functional domains of signalling protein.

The Hippo signalling pathway is an important kinase cascade in the regulation of organ size control and homeostasis. The disruption of Hippo pathway can lead to tumorigenesis^47^. Gene expression signature denoting YAP/TAZ activity has been tightly linked with stem cell signatures in breast cancer^48^. Moreover, YAP is required to sustain self-renewal and tumour initiation of T-ICs in various cancers^37^,^38^. However, targeting core Hippo cascade is presently frustrating, because upstream kinases are negative regulators of YAP, making it incapable to target. Herein, our study demonstrates that inhibition of lncARSR impairs YAP activity and attenuates renal T-ICs propagation. Intriguingly, apart from Hippo pathway wild-type cells as used in this study, we found that lncARSR could also enhance the expansion of T-ICs in Hippo pathway mutant cells, such as ACHN cells (data not show), indicating the involvement of other mechanisms. Thus, lncARSR might serve as a potential therapeutic target for RCCs with aberrant YAP activity in wild-type Hippo signalling.

YAP expression or nuclear accumulation has been reported to correlate with poor patient outcome in several types of cancer^49^. Consistently, our results showed that either high lncARSR expression or nuclear YAP enrichment correlated with poor prognosis of RCC patients. Accumulating evidence indicates that appropriate combination of different markers might be more accurate than a single marker in prognosis evaluation. Herein, we reported that the combination of high lncARSR expression and nuclear YAP accumulation predicted worse prognosis than either marker alone, suggesting a more accurate combinational marker to evaluate the prognosis of RCC patients.

In conclusion, our findings provide insight into the lncARSR/YAP axis as potential therapeutic target against renal T-ICs and powerful predictor for poor prognosis of RCC patients.

## Methods

### Cell lines and reagents

The human RCC cell lines (A498, 769P, Ketr-3, Caki-1, Caki-2) and human embryonic kidney (HEK-293T) cells were purchased from ATCC (Rockville, MD, USA) and cultured in DMEM (Gibco, USA) supplemented with 10% fetal bovine serum (Gibco, USA). The primary ccRCC cell line 771 was established from a ccRCC patient and cultured in DMEM containing 10% fetal bovine serum. EGF and bFGF were purchased from Peprotech (USA), B27 and insulin was purchased from Invitrogen (USA) and Verteporfin was purchased from R&D (#5305, USA).

### RCC patients and clinical specimens

All patient samples were collected from the Department of Urology, Changzheng Hospital with written informed consent. The ethical approval was granted from Committees for Ethical Review in Second Military Medical University. This study involved two cohorts of ccRCC patients, cohort 1, 105 patients and cohort 2, 205 patients. All patients received no previous therapy and were followed until May 2015. All RCC samples are clear cell RCC which has been diagnosed by two pathologists, blinded to the clinical data. lncARSR levels in ccRCC and adjacent tissues were determined by qRT–PCR in cohort 1 and by locked nucleic acid (LNA) ISH on tissue microarray slides in cohort 2.

### *In vivo* xenograft

All experiments involving mice were undertaken in accordance with the National Institute of Health Guide for the Care and Use of Laboratory Animals and the approval of the Institutional Animal Care and Use Committee (IACUC) at Second Military Medical University. Four- to six-week-old male athymic BALB/c nude mice (SIPPR-BK Experimental Animal Co. China) were housed and fed in standard pathogen-free conditions.

For *in vivo* limiting dilution assay, RCC cells were mixed with Matrigel (BD) at a ratio of 1:1 and injected subcutaneously at various cell doses per mouse. Kinetic of tumour formation was evaluated per week for 8 weeks. Frequency of T-ICs was determined using ELDA software (http://bioinf.wehi.edu.au/software/elda/index.html) provided by the Walter and Eliza Hall Institute^50^.

For lung metastasis model, 2 × 10^6^ single cells were injected into the tail vein of nude mice. Mice were killed 12 weeks after inoculation and consecutive sections of the whole lung were subjected to haematoxylin-eosin staining. All of the metastatic lesions in lung were calculated microscopically to evaluate the development of pulmonary metastasis.

### LNA-based *in situ* hybridization

LNA ISH was performed by using miRCURY LNA microRNA ISH Optimization Kit (Exiqon, Denmark) as previously reported^51^ with minor modifications. Briefly, the sections were deparaffinized and then deproteinated using proteinase K (15 μg ml^−1^, Roche) for 10 min at 37 °C. The endogenous peroxidases were inactivated in 1% H_2_O_2_ for 30 min, and sections were pre-hybridized at 62 °C for 30 min in formamide-free Exiqon ISH buffer (Exiqon, Denmark) and hybridized with DIG-labelled LNA probes for lncARSR (5′-AGGTTGTCTGAAGTTGGAGTT-3′, 50 nM, Exiqon, Denmark) at 62 °C overnight. Slides are then stringently washed, incubated with alkaline phosphatase-conjugated anti-DIG Fab fragments (Roche, USA) for 60 min and then detected by NBT/BCIP reagent (Invitrogen, USA). Sections were lastly counterstained with nuclear fast red staining solution (Sigma Chemical Co, USA). High-resolution images were captured with an Aperio Scan Scope AT Turbo (Aperio, USA) equipped with Aperio Image Scope software (Aperio, USA). Assessment of the staining was based on the staining intensity and the percentage of positively stained cells using Image-Pro Plus 6.0 software (Media Cybernetics, Inc., USA). The median signal of lncARSR positive staining was defined as cutoff value.

### Fluorescence *in situ* hybridization

FISH was performed as previously described^52^. The ISH signals were detected using the tyramide signal amplification system (PerkinElmer, USA) and analysed with a fluorescence microscope (IX70, Olympus, Japan).

### Immunocytochemistry

RCC cells were plated in 12-well plates at 30% confluence and allowed to grow for 24 h. Then, the cells were fixed with 10% paraformaldehyde solution for 15 min at room temperature, permeabilized with 0.4% Triton X-100 in PBS for 5 min, and then blocked with 1% BSA in PBS for 1 h at 37 °C. The blocked cells were incubated with anti-FLAG antibody (1:100, Merck Millipore) and anti-YAP antibody (1:100, Cell Signaling Technology) overnight at 4 °C, followed by incubation with Alexa Fluor 488-conjugated anti-mouse IgG antibody and Alexa Fluor 555-conjugated anti-rabbit IgG antibody (1:100, Invitrogen, Carlsbad, CA) for 2 h. Nuclear staining of cells was conducted using 4,6-diamidino-2-phenylindole (DAPI). Representative images were acquired using an fluorescence microscope (IX70, Olympus, Japan).

### Immunohistochemistry

Paraffin-embedded sections were deparaffinized and rehydrated, followed by antigen retrieval. After primary and secondary antibody incubation, the slides were incubated with diaminobenzidine (DAB) (Dako, USA) and counterstained with hematoxylin (Sigma Chemical Co, USA).

Immunohistochemistry of YAP 1:100, (Cell Signaling Technology) was scored by determining the percentage of positive tumour cells (<25% focal, 25–50% moderate and 51–100% diffuse), and their staining intensity (0 negative, 1+ weak, 2+ moderate and 3+ strong, see Supplementary Fig. 7d). Nuclear YAP samples were defined by at least moderate (2+) nuclear staining in ≥25% of tumour cells. Cytoplasmic YAP samples were defined by no visible nuclear staining or weak (1+) nuclear staining.

### Plasmids construction

The cDNA of lncARSR was amplified by using primeSTAR HS DNA polymerase (Takara, China) and subcloned into the KpnI and XhoI sites of pcDNA3.1 vector, and termed pcDNA3.1-lncARSR. The full-length lncARSR, 5′–3′ (1–310) of lncARSR, and 5′–3′ (282–591) of lncARSR were constructed by PCR-based amplification from pcDNA3.1-lncARSR plasmid and subcloned into pSPT19 vector, and termed pSPT19-lncARSR, pSPT19-lncARSR (1–310) or pSPT19-lncARSR (282–591), respectively. pLenti-lncARSR were constructed by PCR-based amplification from pcDNA3.1-lncARSR plasmid and subcloned into pLenti-III vector (Invitrogen, USA). lncARSR-specific shRNA oligos were synthesized by Sangon Co. Ltd (Shanghai). After annealing, double-strand oligos were inserted to lentiviral pLKO.1-Puro vector (Addgene). To produce lentivirus, HEK-293T cells were co-transfected with the lentiviral vector described above and packaging vectors psPAX2 and VSVG using jetPEI (PolyPlus Transfection, France).

The plasmid flag-YAP was kindly provided by professor Fen-yong Sun (Department of Clinical Laboratory Medicine, Shanghai Tenth People's Hospital of Tongji University, Shanghai, China). The plasmids YAP luciferase reporter (8xGTIIC-luciferase, #34615), pCMV-flag-YAP-5SA (#27371), pCMV-flag-YAP-5SA/S94A (#33103), pLKO. 1-shYAP (#42540), pcDNA-HA-MST2 (#33098) and pcDNA-Lats1 (#41156) were purchased from Addgene. The YAP truncation mutants were constructed by PCR-based amplification from flag-YAP plasmid.

All constructs were confirmed by DNA sequencing. Sequences of primers used for plasmid construction in this study were listed in Supplementary Table 12.

### Cell transfection

Transfection of plasmids was performed by using jetPEI (PolyPlus Transfection, France). Transfection of siRNA (100 nM, GenePharma, China) was performed by using Lipofectamine RNAiMAX (Invitrogen, USA). Sequences of siRNA against specific targets were listed in Supplementary Table 13.

### RNA isolation and RT–PCR analysis

Total RNA was extracted by TRIzol (Invitrogen, USA). Real-time quantitative PCR was performed on triplicate samples in a reaction mix of SYBR Green (Takara, China) by ABI 7900HT Fast Real-Time PCR System (Applied Biosystems, USA). The expression of indicated genes was normalized to endogenous reference control β-actin by using 2^−ΔΔCt^ method. Sequences of primers used for qRT–PCR in this study were listed in Supplementary Table 14.

### RNA pull-down

RNA pull-down was performed as previously described^14^,^53^. Briefly, biotin-labelled RNA was transcribed *in vitro* using Biotin RNA Labelling Mix (Roche, USA) and T7 or SP6 RNA polymerase (Promega, USA), respectively, treated with RNase-free DNase I (Roche, USA) and then purified with RNeasy Mini Kit (Qiagen, Germany). Biotinylated RNA was incubated with cytoplasmic extract of RCC cells at room temperature for 1 h. Washed streptavidin agarose beads (Invitrogen, USA) were added to each binding reaction and incubated at room temperature for 1 h. Precipitates were washed for three times, boiled in SDS buffer and subjected to SDS–polyacrylamide gel electrophoresis. Specific bands were excised and analysed by mass spectrometry or subjected to western blot detection.

### Western blot

Cell lysates or retrieved proteins were analysed by immunoblot with primary antibodies and IRDye 800 CW-conjugated secondary antibody (Rockland Immunochemicals, USA). The intensity of the fluorescence was scanned by Odyssey fluorescence scanner system (Li-Cor Biosciences, USA). Primary antibodies used in this study are listed in Supplementary Table 15.

### RNA immunoprecipitation

RIP was conducted by EZ-Magna RIP RNA-Binding Protein Immunoprecipitation Kit (Millipore, USA). The RNA fraction precipitated by RIP was analysed by real-time quantitative PCR. Total RNAs (input) and IgG controls were assayed simultaneously to demonstrate that the detected signals were the result of RNAs specifically binding to indicated antibody.

### Chromatin immunoprecipitation

ChIP was conducted by using EZ-ChIP Kit (Millipore, USA). ChIP–re-ChIP was conducted as previously described^54^. Pooled eluants were diluted to a final SDS concentration of 0.1% and incubated with fresh antibody-bound beads for the second immunoprecipitation. Fold enrichment was quantified using quantitative RT–PCR and calculated as a percentage of input chromatin (% Input). Sequences of primers used for ChIP–quantitative PCR (qPCR) in this study were listed in Supplementary Table 16.

### Electrophoretic mobility shift assay

EMSA was performed using a LightShift Chemiluminescent RNA EMSA Kit (#20158, Thermo Scientific, USA) according to manufacturer's instruction. The biotin-labelled RNA transcripts were *in vitro* transcribed with the Biotin RNA Labeling Mix as described in RNA pull-down assay. The human YAP recombinant protein was purchased from Abcam (ab#132459).

### Luciferase reporter assay

A498 cells were co-transfected with YAP/TAZ luciferase reporter and pcDNA3.1-ARSR using Lipofectamine 2000 (Invitrogen). Each group was run in triplicate in 48-well plates. The luciferase activity was detected by Synergy 2 Multidetection Microplate Reader (BioTek Instruments, Inc.) after 48 h of transfection. Renilla luciferase activity was normalized against Firefly luciferase activity.

### *In vitro* phosphorylation assay

A498 cells were co-transfected with plasmids MYC-LATS1 and HA-MST2. After 48 h of transfection, cells were lysed with IP buffer and immunoprecipitated with anti-MYC antibody. The immmunoprecipitates were washed three times with IP buffer, and then washed once with kinase buffer (20 mM Tris-HCl (pH 7.5), 15 mM MgCl_2_, 5 mM EGTA, 100 mM NaCl and 1 mM DTT). One microgram of recombinant human YAP was incubated with the immunoprecipitated LATS1 and biotin-labelled RNA in 25 μl kinase buffer containing 100 μM ATP at 30 °C for 1 h with gentle shaking. The reactions were terminated with SDS-loading buffer and YAP phosphorylation was detected by western blot.

### Spheres formation assay

One thousand single cells were seeded into 96-well Ultra-Low Attachment Microplates (Corning, USA) in serum-free DMEM/F12 (Invitrogen, USA), supplemented with B27 (1:50, Invitrogen), 20 ng ml^−1^ EGF (Peprotech), 10 ng ml^−1^ bFGF (Invitrogen), and 4 μg ml^−1^ insulin (Sigma)^55^. Spheres were photographed and counted 7 days after seeding (primary spheres). To propagate spheres *in vitro*, spheres were collected by centrifugation and trypsinized with 0.25% trypsin to obtain single cell, and equal number of cells were then seeded into ultra-low attachment plate (secondary spheres).

### *In vitro* limiting dilution assay

RCC cells were seeded into 96-well Ultra-Low Attachment Microplates (Corning, USA) at various cell doses and incubated under spheres forming conditions for 7 days. Based on the frequency of wells with spheres forming, the proportion of tumour-initiating cells was determined using Poisson distribution statistics and the LCalc Version 1.1 software program (Stem Cell Technologies, Inc. Vancouver, Canada).

### Flow cytometry

RCC cells were collected and washed with PBS. RCC cells were incubated with indicated antibody or isotype control antibody for 30 min on ice in the dark. Samples were analysed by FACS apparatus MoFlo XDP (Beckman Coulter, USA).

### Data analysis

All statistical analyses in this study were performed with SPSS 16.0 software (SPSS Inc., USA). Data were presented as ‘mean±s.d.'. The significance of mean values between two groups was analysed by two-tailed Student's *t*-test. Spearman's correlation analysis was performed to determine the correlation between two variables. Pearson chi-square test acted to analyse the clinical variables. Kaplan–Meier survival analysis was utilized to compare ccRCC patient survival based on dichotomized lncARSR expression by log-rank test. Cox proportional hazards regression analyses were utilized to analyse the effect of clinical variables on patient survival. A *P* value <0.05 was considered significant.

### Data availability

The gene expression profiling of generated sunitinib-resistant RCC cell lines have been deposited in GEO with the accession code GSE69535. The mass spectrum data that support the findings of this study are included in Supplementary Data 1. The authors declare that all other relevant data supporting the findings of this study are available on request.

## Additional information

**How to cite this article:** Qu, L. *et al*. A feed-forward loop between lncARSR and YAP activity promotes expansion of renal tumour-initiating cells. *Nat. Commun.* 7:12692 doi: 10.1038/ncomms12692 (2016).

## Supplementary Material

Supplementary Information Supplementary Figures 1-8 and Supplementary Tables 1-16.

Supplementary Data 1 Contains the raw data of mass spectrometry analysis of the protein band pulled down by lncARSR.
