# Supplementary material for: A feed-forward loop between lncARSR and YAP activity promotes expansion of renal tumour-initiating cells
Source: Nat Commun. 2016 Nov 25;7:12692. doi: 10.1038/ncomms12692 (PMC5133634; doi:10.1038/ncomms12692)
Supplement: Supplementary Information — Supplementary Figures 1-8 and Supplementary Tables 1-16. [file ncomms12692-s1.pdf]

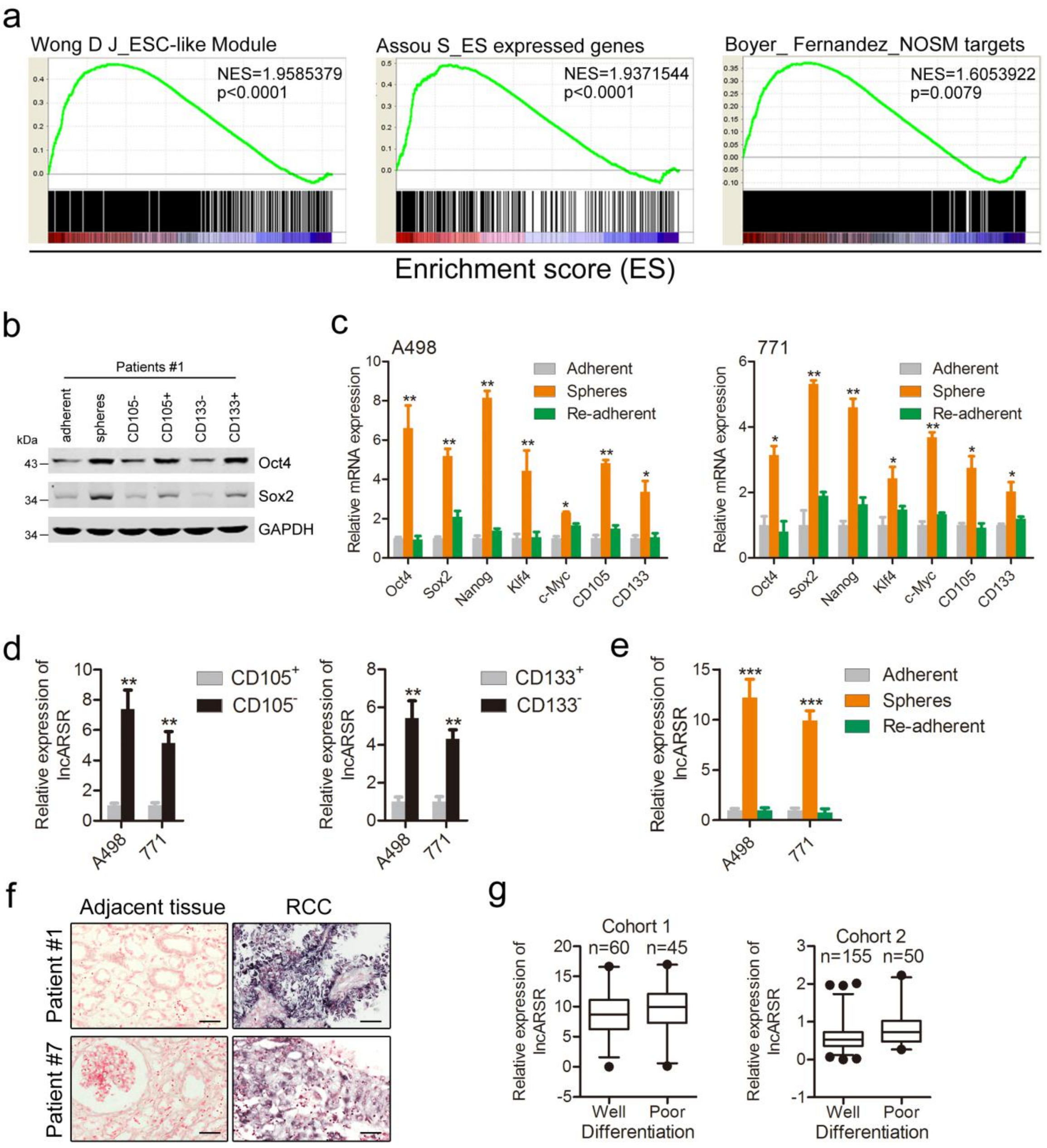
a
Wong D J_ESC-like Module
NES=1.9585379
p<0.0001
Assou S_ES expressed genes
NES=1.9371544
p<0.0001
Boyer_ Fernandez_NOSM targets
NES=1.6053922
p=0.0079
Enrichment score (ES)
b
Patients #1
adherent
spheres
CD105-
CD105+
CD133-
CD133+
kDa
Oct4
Sox2
GAPDH
c
A498
Relative mRNA expression
Adherent
Spheres
Re-adherent
Oct4
Sox2
Nanog
Klf4
c-Myc
CD105
CD133
771
Sphere
d
Relative expression of lncARSR
CD105+
CD105-
CD133+
CD133-
A498
771
e
f
Adjacent tissue
RCC
Patient #1
Patient #7
g
Cohort 1
n=60
n=45
Cohort 2
n=155
n=50
Well
Poor
Differentiation

**Supplementary Figure 1. lncARSR is upregulated in T-ICs and predicts poor prognosis**

(a) Gene set enrichment analysis using three stem cell-like gene signatures. NES, normalized enrichment score. (b) Western blot analysis of indicated proteins in enriched T-Ics from primary RCC cells. (c) qRT-PCR analysis of indicated mRNAs in RCC adherent, spheres and re-adherent cells ($n$ = 3). (d) qRT-PCR analysis of lncARSR in MACS sorted $CD105^{+}$ (left) or $CD133^{+}$ (right) RCC cells relative to negative cells ($n$ = 3). (e) qRT-PCR analysis of lncARSR in RCC adherent, spheres and re-adherent cells ($n$ = 3). (c-e) Data are represented as mean ± s.d.; $*p < 0.05$, $**p < 0.01$ and $***p < 0.001$; two-tailed Student's *t*-test. (f) Representative images of lncARSR expression detected by RNA ISH (in situ hybridization) in cohort 2. Scale bar = 50 µm. (g) Comparison of lncARSR levels in well or poorly differentiated RCC tissues in cohorts 1 (left, $p = 0.013$, Mann–Whitney *U*-test) and cohort 2 (right, $p < 0.001$, Mann–Whitney *U*-test). The horizontal lines in the box plots represent the median, the boxes represent the interquartile range, and the whiskers represent the 2.5th and 97.5th percentiles.

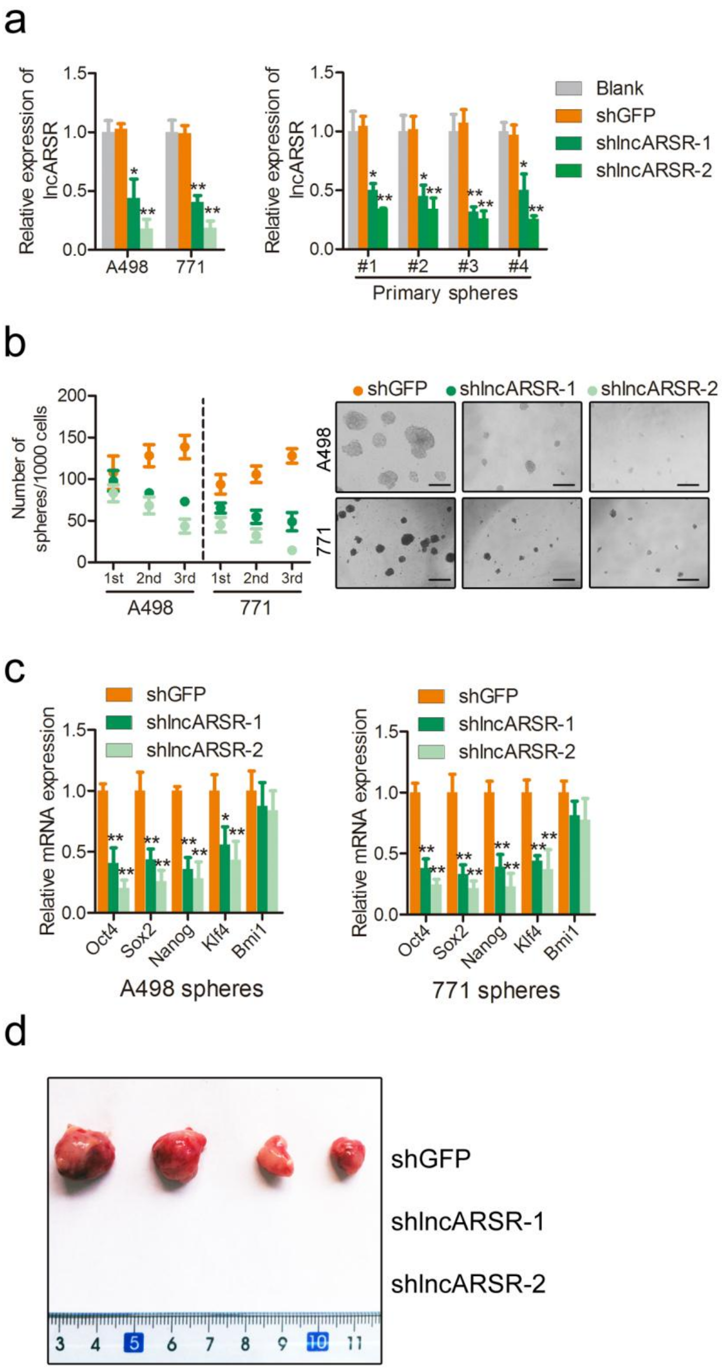

a
Relative expression of lncARSR
A498
771
#1
#2
#3
#4
Primary spheres
Blank
shGFP
shlncARSR-1
shlncARSR-2
b
Number of spheres/1000 cells
1st
2nd
3rd
A498
771
c
Relative mRNA expression
Oct4
Sox2
Nanog
Klf4
Bmi1
A498 spheres
771 spheres
d
shGFP
shlncARSR-1
shlncARSR-2

**Supplementary Figure 2. lncARSR is required for the maintenance of renal T-ICs**

(a) Left: qRT-PCR analysis of lncARSR expression in lncARSR-knockdown and control RCC cells (*n* = 3). Right: qRT-PCR analysis of lncARSR expression in lncARSR-knockdown and control primary RCC cells (*n* = 3). Data are represented as mean ± s.d.; $*p < 0.05$ and $**p < 0.01$; two-tailed Student's *t*-test. (b) Spheres formation assay of lncARSR-knockdown and control RCC cells (*n* = 3). The number of primary, secondary and tertiary passaged spheres was counted after 7 days (left). Representative images of spheres are shown (right). Scale bar = 200 μm. (c) qRT-PCR analysis of indicated mRNAs in lncARSR-knockdown and control RCC spheres (*n* = 3). Data are represented as mean ± s.d.; $*p < 0.05$ and $**p < 0.01$; two-tailed Student's *t*-test. (d) Subcutaneous xenograft assay of $1\times10^4$ RCC cells derived from lncARSR-knockdown and control xenografts for 2 months (*n* = 4 per group).

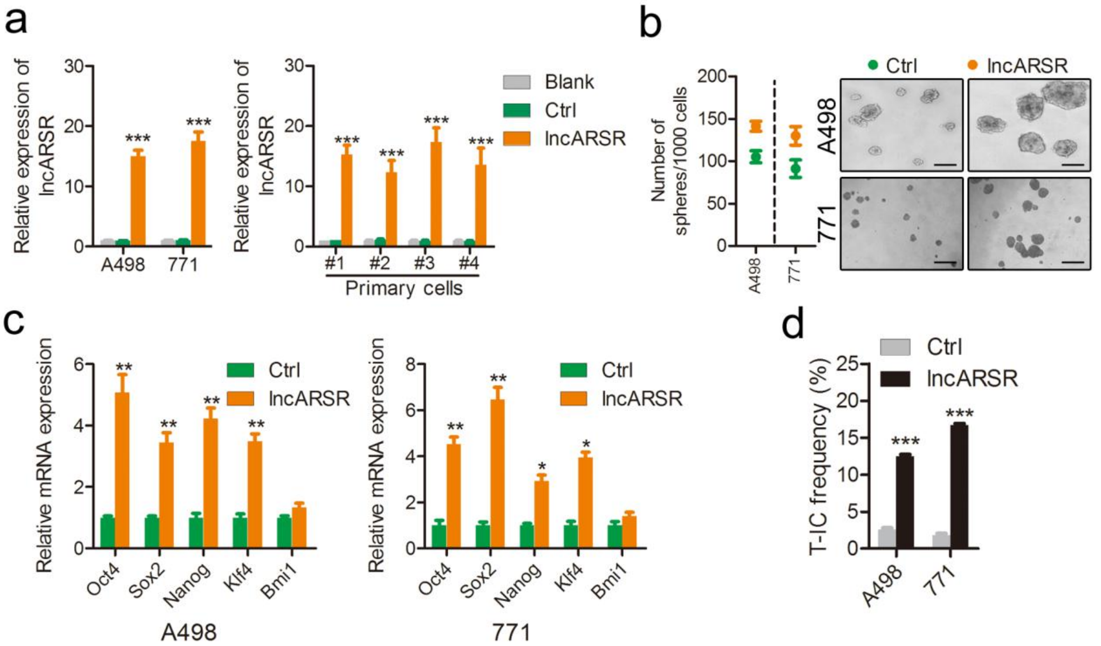


**Supplementary Figure 3. lncARSR promotes renal T-ICs expansion**

(a) Left: qRT-PCR analysis of lncARSR expression in lncARSR-overexpressing and control RCC cells ($n$ = 3). Right: qRT-PCR analysis of lncARSR expression in lncARSR-overexpressing and control primary RCC cells ($n$ = 3). (b) Spheres formation assay of lncARSR-overexpressing and control RCC cells ($n$ = 3). The number of spheres was counted after 7 days (left). Representative images of spheres are shown (right). Scale bar = 200 μm. (c) qRT-PCR analysis of indicated mRNAs in lncARSR-overexpressing and control RCC cells ($n$ = 3). (d) In vitro limiting dilution assay of lncARSR-overexpressing and control RCC cells. The results were shown as natural logarithm of the proportion of T-ICs. (a, c and d) Data are represented as mean ± s.d.; *$p$ < 0. 05, **$p$ < 0. 01 and ***$p$ < 0. 001; two-tailed Student's $t$-test.

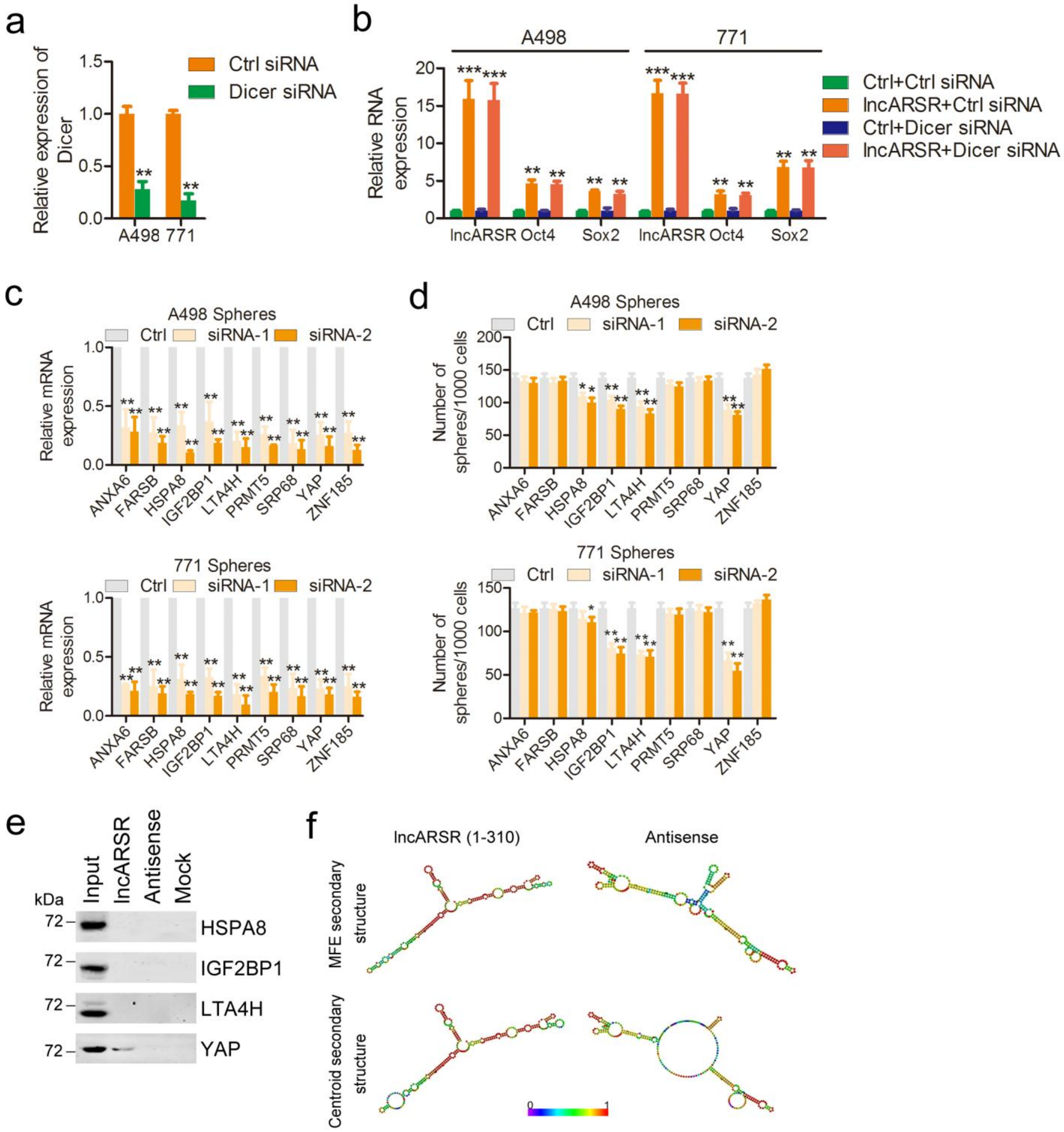

a
Relative expression of Dicer
Ctrl siRNA
Dicer siRNA
A498
771
b
A498
771
Relative RNA expression
Ctrl+Ctrl siRNA
lncARSR+Ctrl siRNA
Ctrl+Dicer siRNA
lncARSR+Dicer siRNA
lncARSR
Oct4
Sox2
c
A498 Spheres
771 Spheres
Ctrl
siRNA-1
siRNA-2
Relative mRNA expression
ANXA6
FARSB
HSPA8
IGF2BP1
LTA4H
PRMT5
SRP68
YAP
ZNF185
d
Number of spheres/1000 cells
e
kDa
Input
lncARSR
Antisense
Mock
HSPA8
IGF2BP1
LTA4H
YAP
f
lncARSR (1-310)
Antisense
MFE secondary structure
Centroid secondary structure

**Supplementary Figure 4. lncARSR physically interacts with YAP**

(a) qRT-PCR analysis of Dicer expression in RCC cells transfected with Dicer siRNA after 48h ($n$ = 3). (b) qRT-PCR analysis of indicated RNAs in RCC cells transfected with lncARSR plasmid and Dicer siRNA ($n$ = 3). (c) Verification of interference efficiency of siRNA for indicated mRNAs in RCC spheres by qRT-PCR ($n$ = 3). (d) Spheres formation assay of RCC spheres transfected with indicated siRNAs ($n$ = 3). The number of spheres was counted after 7 days (left). Representative images of spheres are shown (right). Scale bar = 200 μm. (a-d) Data are represented as mean ± s.d.; *$p$ < 0. 05, **$p$ < 0. 01 and ***$p$ < 0. 001; two-tailed Student's $t$-test. (e) Western blot analysis of indicated proteins in RNA pull-down precipitates retrieved by biotin-labeled lncARSR or antisense RNA from the cytoplasmic lysates of A498 spheres. (f) Secondary structure prediction of 5' segment of lncARSR and its antisense sequence based on minimum free energy (MFE) and partition function (http://rna.tbi.univie.ac.at/). Color scale indicates the prediction confidence for each base with red indicating strong confidence.

Data are shown as means ± s.d. *$p$ < 0. 05; **$p$ < 0. 01; and ***$p$ < 0. 001.

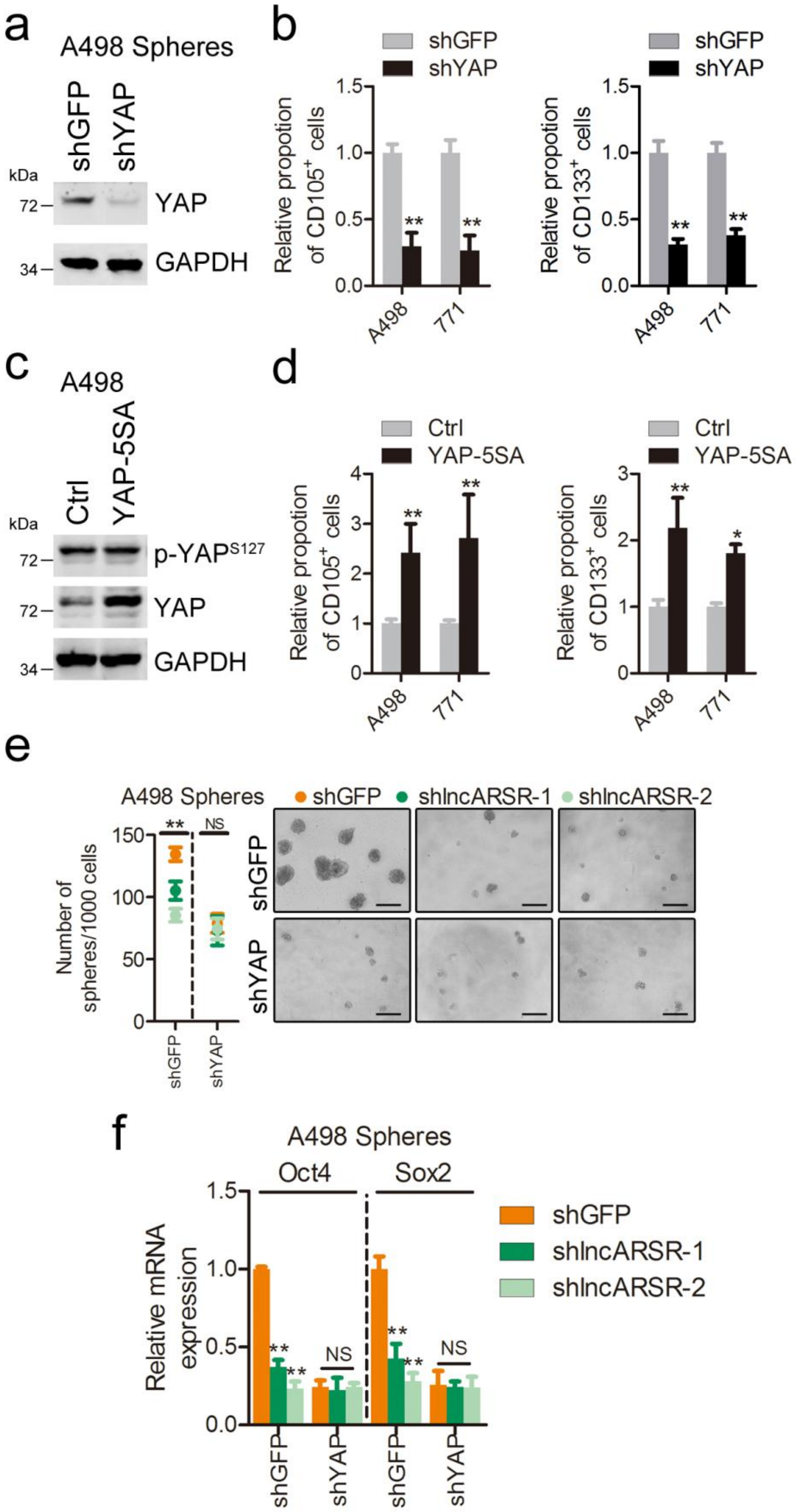
a
A498 Spheres
shGFP
shYAP
kDa
72
YAP
34
GAPDH
b
shGFP
shYAP
Relative propotion of CD105+ cells
Relative propotion of CD133+ cells
A498
771
c
A498
Ctrl
YAP-5SA
p-YAPS127
YAP
GAPDH
d
Ctrl
YAP-5SA
e
A498 Spheres
shGFP
shlncARSR-1
shlncARSR-2
Number of spheres/1000 cells
NS
shYAP
f
A498 Spheres
Oct4
Sox2
Relative mRNA expression

**Supplementary Figure 5. YAP is responsible for lncARSR-mediated T-IC properties**

(a) Western blot analysis of YAP in YAP-knockdown and control A498 spheres. (b) Flow cytometric analysis of the proportion of CD105+ (left) or CD133+ (right) cells in YAP-knockdown and control RCC cells ($n$ = 3). (c) Western blot analysis of YAP in YAP-5SA-overexpressing and control A498 cells. (d) Flow cytometric analysis of the proportion of CD105+ (left) or CD133+ (right) cells in YAP-5SA-overexpressing and control RCC cells ($n$ = 3). (e) Spheres formation assay of A498 spheres transfected with indicated plasmids ($n$ = 3). The number of spheres was counted after 7 days (left). Representative images of spheres are shown (right). Scale bar = 200 μm. (f) qRT-PCR analysis of Oct4 and Sox2 in A498 spheres transfected with indicated plasmids after 48h ($n$ = 3). (b, d-f) Data are represented as mean ± s.d.; *$p$ < 0. 05 and **$p$ < 0. 01; two-tailed Student's $t$-test.

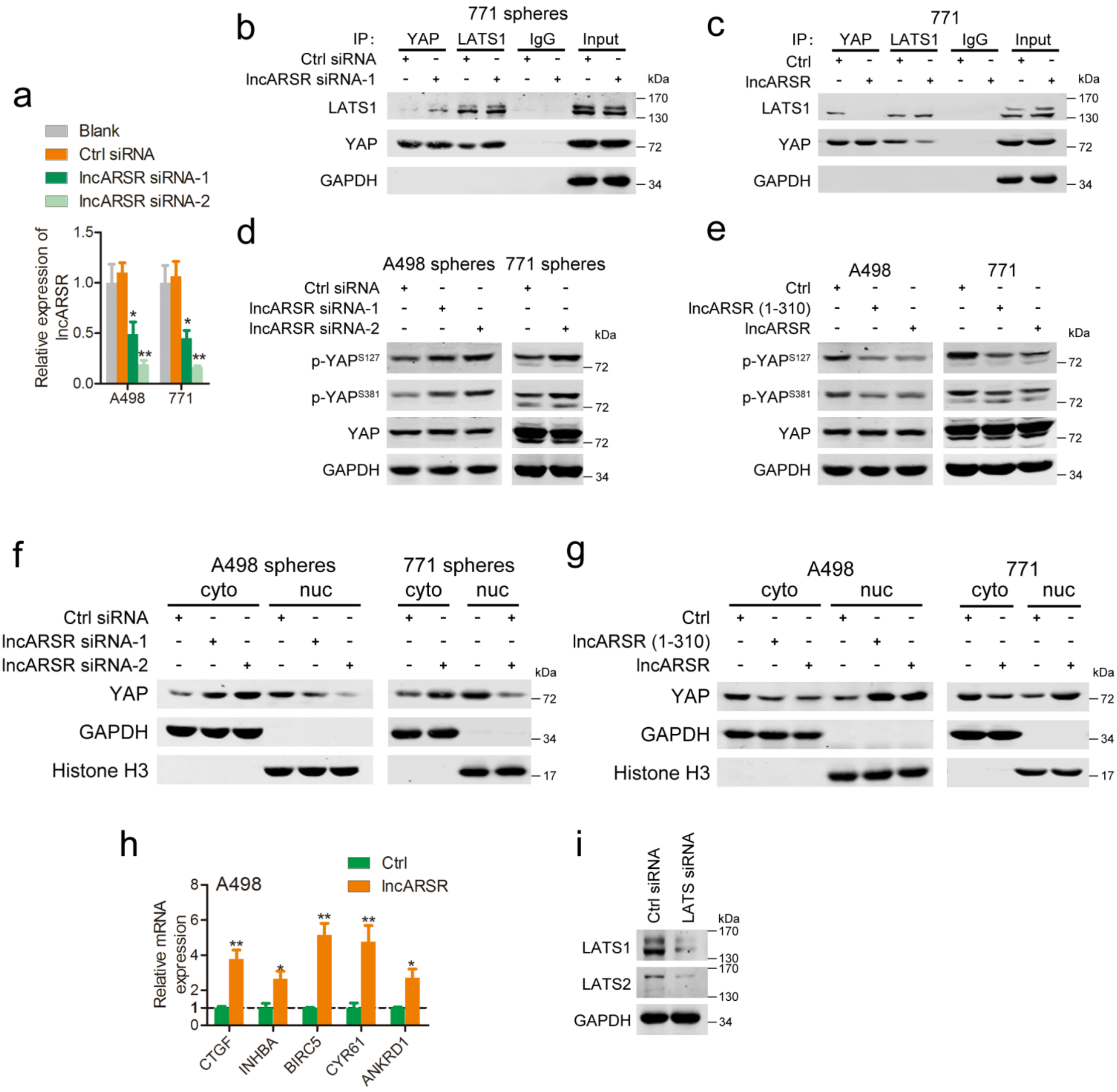
a
Blank
Ctrl siRNA
lncARSR siRNA-1
lncARSR siRNA-2
Relative expression of lncARSR
1.5
1.0
0.5
0.0
A498
771
b
771 spheres
IP:
YAP
LATS1
IgG
Input
Ctrl siRNA
lncARSR siRNA-1
kDa
LATS1
YAP
GAPDH
170
130
72
34
c
771
IP:
YAP
LATS1
IgG
Input
Ctrl
lncARSR
kDa
LATS1
YAP
GAPDH
d
A498 spheres
771 spheres
Ctrl siRNA
lncARSR siRNA-1
lncARSR siRNA-2
kDa
p-YAPS127
p-YAPS381
YAP
GAPDH
e
A498
771
Ctrl
lncARSR (1-310)
lncARSR
kDa
p-YAPS127
p-YAPS381
YAP
GAPDH
f
A498 spheres
771 spheres
cyto
nuc
Ctrl siRNA
lncARSR siRNA-1
lncARSR siRNA-2
kDa
YAP
GAPDH
Histone H3
17
g
A498
771
cyto
nuc
Ctrl
lncARSR (1-310)
lncARSR
kDa
YAP
GAPDH
Histone H3
h
A498
Ctrl
lncARSR
Relative mRNA expression
8
6
4
2
1
0
CTGF
INHBA
BIRC5
CYR61
ANKRD1
i
Ctrl siRNA
LATS siRNA
kDa
LATS1
LATS2
GAPDH

**Supplementary Figure 6. lncARSR blocks LATS1-mediated YAP phosphorylation**

(a) qRT-PCR analysis of lncARSR expression in lncARSR-knockdown and control RCC cells ($n$ = 3). Data are represented as mean ± s.d.; *$p$ < 0. 05 and **$p$ < 0. 01; two-tailed Student's $t$-test. (b) Coimmunoprecipitation of YAP and LATS1 in lysates of lncARSR-knockdown and control 771 spheres. GAPDH acted as a loading control. (c) Coimmunoprecipitation of YAP and LATS1 in lysates of lncARSR-overexpressing and control 771 cells. GAPDH acted as a loading control. (d) Western blot analysis of YAP in lncARSR-knockdown and control RCC spheres. (e) Western blot analysis of YAP in RCC cells with lncARSR overexpression or 5′ segment (nucleotides 1-310) overexpression. (f) Western blot analysis of YAP in subcellular fractions of lncARSR-knockdown and control RCC spheres. GAPDH and Histone H3 acted as cytoplasm and nucleus marker respectively. (g) Western blot analysis of YAP in subcellular fractions of RCC cells with lncARSR overexpression or 5′ segment (nucleotides 1-310) overexpression. GAPDH and Histone H3 acted as cytoplasm and nucleus marker respectively. (h) qRT-PCR analysis of indicated mRNAs in lncARSR-overexpressing and control A498 cells ($n$ = 3). Data are represented as mean ± s.d.; *$p$ < 0. 05 and **$p$ < 0. 01; two-tailed Student's $t$-test. (i) Western blot analysis of LATS1 and LATS2 in A498 cells transfected with LATS siRNA mix after 48h.

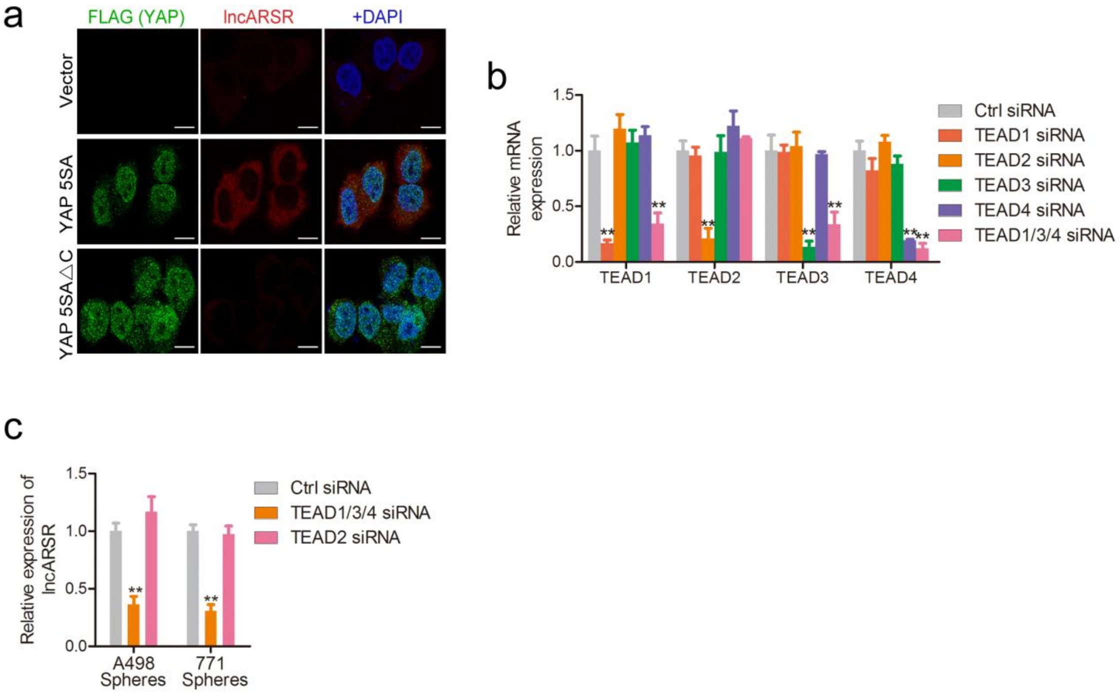


**Supplementary Figure 7. YAP/TEAD complex transactivates lncARSR**

(a) RNA FISH analysis of lncARSR and immunofluorescence detection of YAP in A498 cells transfected with Flag-YAP-5SA plasmid or Flag-YAP-5SAΔC plasmid after 48h. Scale bars = 10 μm. (b) Verification of interference efficiency of TEADs siRNA for indicated mRNAs in RCC spheres by qRT-PCR (*n* = 3). (c) qRT-PCR analysis of lncARSR in RCC spheres transfected with indicated siRNA after 48h (*n* = 3). (b and c) Data are represented as mean ± s.d.; $^{**}p < 0.01$; two-tailed Student's *t*-test.

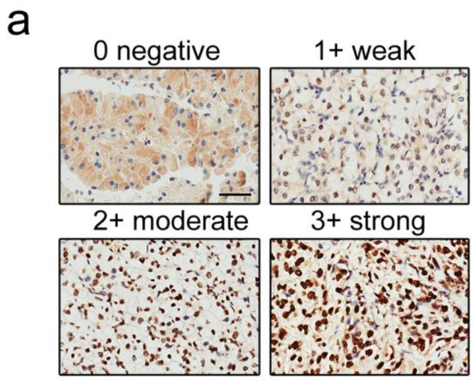


**Supplementary Figure 8. Combining lncARSR and YAP exhibits improved prognostic value**

(a) Representative images of different nuclear YAP staining intensity in RCC tissues in Cohort 2. Scale bar = 50 μm.

**Supplementary Table 1. Clinical Characteristics of RCC Patients**

| **Variables** | **Cohort 1 (*n* = 105)** | **Cohort 2 (*n* = 205)** |
|---|---|---|
| **Gender (%)** | | |
| Male | 65 (61.9) | 139 (67.8) |
| Female | 40 (38.1) | 66 (32.2) |
| **Age (%)** | | |
| ≤60 | 69 (65.7) | 133 (64.9) |
| ＞60 | 36 (34.3) | 72 (35.1) |
| **Tumor size (%)** | | |
| ≤4 cm | 45 (42.9) | 86 (41.9) |
| ＞4 cm | 60 (57.1) | 119 (58.1) |
| **Furman grade (%)** | | |
| Ⅰ and Ⅱ | 60 (57.1) | 155 (75.6) |
| Ⅲ and Ⅳ | 45 (42.9) | 50 (24.4) |
| **TNM stage (%)** | | |
| Ⅰ and Ⅱ | 62 (87.6) | 142 (69.3) |
| Ⅲ and Ⅳ | 43 (12.4) | 63 (30.7) |
| **Tumor thrombus (%)** | | |
| No | 95 (90.5) | 186 (90.7) |
| Yes | 10 (9.5) | 19 (9.3) |
| **Distant metastasis (%)** | | |
| No | 92 (87.6) | 173 (84.4) |
| Yes | 13 (12.4) | 32 (15.6) |

**Supplementary Table 2. Correlation of lncARSR Levels and Clinical Characteristics in Cohort 1**

| Variables | Low lncARSR (*n* = 53) | High lncARSR (*n* = 52) | *p*-value |
|---|---|---|---|
| **Gender** | | | 1.000 |
| Male | 30 | 30 | |
| Female | 23 | 22 | |
| **Age** | | | 0.838 |
| ≤60 | 34 | 35 | |
| ＞60 | 19 | 17 | |
| **Tumor size** | | | 0.238 |
| ≤4cm | 26 | 19 | |
| ＞4cm | 27 | 33 | |
| **Furman grade** | | | 0.031 |
| Ⅰ/Ⅱ | 36 | 24 | |
| Ⅲ/Ⅵ | 17 | 28 | |
| **TNM stage** | | | 0.010 |
| Ⅰ/Ⅱ | 38 | 24 | |
| Ⅲ/Ⅵ | 15 | 28 | |
| **Tumor thrombus** | | | 0.052 |
| No | 51 | 44 | |
| Yes | 2 | 8 | |
| **Distant metastasis** | | | 0.042 |
| No | 50 | 42 | |
| Yes | 3 | 10 | |

**Supplementary Table 3. Correlation of lncARSR Levels and Clinical Characteristics in Cohort 2**

| Variables | Low lncARSR (*n* = 103) | High lncARSR (*n* = 102) | *p*-value |
|---|---|---|---|
| **Gender** | | | 0.296 |
| Male | 66 | 73 | |
| Female | 37 | 29 | |
| **Age** | | | 0.661 |
| ≤60 | 65 | 68 | |
| ＞60 | 38 | 34 | |
| **Tumor size** | | | 0.888 |
| ≤4cm | 44 | 42 | |
| ＞4cm | 59 | 60 | |
| **Furman grade** | | | 0.023 |
| Ⅰ/Ⅱ | 85 | 70 | |
| Ⅲ/Ⅵ | 18 | 32 | |
| **TNM stage** | | | 0.010 |
| Ⅰ/Ⅱ | 80 | 62 | |
| Ⅲ/Ⅵ | 23 | 40 | |
| **Tumor thrombus** | | | 0.008 |
| No | 99 | 87 | |
| Yes | 4 | 15 | |
| **Distant metastasis** | | | 0.002 |
| No | 95 | 78 | |
| Yes | 8 | 24 | |

**Supplementary Table 4. Univariate and Multivariate Analyses of Factors Associated with Overall Survival in Cohort 1**

| Variable | Univariate | Multivariate | | |
|---|---|---|---|---|
| | | **Hazard Ratio** | **95% CI** | ***p*-value** |
| **lncARSR expression** | | | | |
| High vs Low | 0.004 | 3.215 | 1.269-8.145 | 0.014* |
| **Furman grade** | | | | |
| Ⅲ/Ⅵvs Ⅰ/Ⅱ | 0.040 | 1.236 | 0.489-3.125 | 0.655 |
| **TNM** | | | | |
| Ⅲ/Ⅵvs Ⅰ/Ⅱ | 0.006 | 2.015 | 0.771-5.265 | 0.153 |
| **Tumor thrombus** | | | | |
| Yes vs No | 0.047 | 1.222 | 0.419-3.565 | 0.713 |
| **Distant metastasis** | | | | |
| Yes vs No | 0.003 | 2.052 | 0.756-5.570 | 0.158 |

**Supplementary Table 5. Univariate and Multivariate Analyses of Factors Associated with Recurrence in Cohort 1**

| Variable | Univariate | Multivariate | | |
|---|---|---|---|---|
| | | **Hazard Ratio** | **95% CI** | ***p*-value** |
| **lncARSR expression** | | | | |
| High vs Low | 0.001 | 3.073 | 1.476-6.400 | 0.003* |
| **TNM** | | | | |
| Ⅲ/Ⅵvs Ⅰ/Ⅱ | 0.043 | 1.242 | 0.574-2.688 | 0.583 |
| **Distant metastasis** | | | | |
| Yes vs No | 0.003 | 2.736 | 1.279-5.851 | 0.009* |

**Supplementary Table 6. Univariate and Multivariate Analyses of Factors Associated with Overall Survival in Cohort 2**

| Variable | Univariate | Multivariate | | |
|---|---|---|---|---|
| | | **Hazard Ratio** | **95% CI** | ***p*-value** |
| **lncARSR expression** | | | | |
| High vs Low | 0.008 | 2.173 | 1.230-3.837 | 0.007* |
| **TNM** | | | | |
| Ⅲ/Ⅵvs Ⅰ/Ⅱ | 0.009 | 2.186 | 1.079-4.428 | 0.030* |
| **Distant metastasis** | | | | |
| Yes vs No | 0.005 | 2.451 | 1.187-5.061 | 0.015* |

**Supplementary Table 7. Univariate and Multivariate Analyses of Factors Associated with Recurrence in Cohort 2**

| Variable | Univariate | Multivariate | | |
|---|---|---|---|---|
| | | **Hazard Ratio** | **95% CI** | ***p*-value** |
| **lncARSR expression** | | | | |
| High vs Low | 0.002 | 2.023 | 1.213-3.375 | 0.007* |
| **TNM** | | | | |
| Ⅲ/Ⅵvs Ⅰ/Ⅱ | 0.048 | 1.751 | 0.911-3.369 | 0.093 |
| **Tumor thrombus** | | | | |
| Yes vs No | 0.138 | 0.992 | 0.510-1.932 | 0.982 |
| **Distant metastasis** | | | | |
| Yes vs No | 0.004 | 2.793 | 1.393-5.597 | 0.004* |

**Supplementary Table 8. Frequency of T-ICs in lncARSR-knockdown and control sphere-derived RCC cells**

| Cell type inoculated | T-ICs Frequency | | *p*-value |
|---|---|---|---|
| | Estimate | Upper and Lower Limits | Ratio of prop.'s=1 |
| **A498 Sphere** | | | |
| shGFP | 1/3181 | 1/8043-1/1258 | |
| shlncARSR-1 | 1/38490 | 1/94240-1/15720 | <0.0001 |
| shlncARSR-2 | 1/84598 | 1/214144-1/33421 | <0.0001 |
| **771 Sphere** | | | |
| shGFP | 1/7908 | 1/19790-1/3160 | |
| shlncARSR-1 | 1/84598 | 1/214144-1/33421 | 0.0002 |
| shlncARSR-2 | 1/280334 | 1/1115137-1/70473 | <0.0001 |

**Supplementary Table 9. Frequency of T-ICs in lncARSR-overexpressing and control RCC cells**

| Cell type inoculated | T-ICs Frequency | | *p*-value |
|---|---|---|---|
| | Estimate | Upper and Lower Limits | Ratio of prop.'s=1 |
| **A498** | | | |
| Ctrl | 1/460139 | 1/1110523-1/190656 | |
| lncARSR | 1/57086 | 1/138002-1/23614 | 0.0007 |
| **771** | | | |
| Ctrl | 1/1228510 | 1/3410211-1/442564 | |
| lncARSR | 1/122257 | 1/333981-1/44753 | 0.0009 |

**Supplementary Table 10. Frequency of T-ICs in indicated RCC cells**

| Cell type inoculated | T-ICs Frequency | | *p*-value |
|---|---|---|---|
| | Estimate | Upper and Lower Limits | Ratio of prop.'s=1 |
| **A498** | | | |
| Ctrl shGFP | 1/376880 | 1/925194-1/153523 | |
| lncARSR shGFP | 1/31801 | 1/80421-1/12575 | <0.0001 |
| Ctrl shYAP | 1/681902 | 1/1670761-1/278311 | 0.318 |
| lncARSR shYAP | 1/384893 | 1/942399-1/157197 | 0.971 |

**Supplementary Table 11. Correlation of Nuclear YAP and lncARSR Levels in Cohort 2**

| | **Low lncARSR** | **High lncARSR** | ***p*-value** |
|---|---|---|---|
| **cyto-YAP** | 79 | 38 | p < 0.001 |
| **nuc-YAP** | 24 | 64 | |

**Supplementary Table 12. Sequences of Primers Used for Plasmid Construction**

| | | |
|---|---|---|
| pcDNA3.1 -lncARSR | Forward (5’-3’) | GGGGTACCACCCCCGGAGGCACTCG |
| | Reverse (5’-3’) | CCGCTCGAGTTTTTTTTTTGTTTTATCAAA GGAATTTA |
| pSPT19 -lncARSR | Forward (5’-3’) | GCTCTAGAACCCCCGGAGGCACTCG |
| | Reverse (5’-3’) | CCCAAGCTTTTTTTTTTTTGTTTTATCAAAG GAATTTA |
| pSPT19 -lncARS R -5’ (1-310) | Reverse (5’-3’) | CCGCTCGAG TGAGACCAGCCAGTTGAGTTGG |
| pSPT19 -lncARSR -3’(282-591) | Forward (5’-3’) | GCCC AAGCTT CTATACCCCAACTCAACTGGCTGG |
| shlncARSR-1 | Sense (5’-3’) | CCGGGAGCATGAAGAACTCCAACTTCTCG AGAAGTTGGAGTTCTTCATGCTCTTTTT |
| | Antisense (5’-3’) | AATTAAAAAGAGCATGAAGAACTCCAACT TCTCGAGAAGTTGGAGTTCTTCATGCTC |
| shlncARSR-2 | Sense (5’-3’) | CCGGGCATGAAGAACTCCAACTTCACTCG AGTGAAGTTGGAGTTCTTCATGCTTTTT |
| | Antisense (5’-3’) | AATTAAAAAGCATGAAGAACTCCAACTTC ACTCGAGTGAAGTTGGAGTTCTTCATGC |
| shGFP | Sense (5’-3’) | 5'-CCGGGCAAGCTGACCCTGAAGTTCATCT CGAGATGAACTTCAGGGTCAGCTTGCTTT TTG-3' |
| | Antisense (5’-3’) | 5'-AATTCAAAAAGCAAGCTGACCCTGAAG TTCATCTCGAGATGAACTTCAGGGTCAGCT TGC-3' |
| YAP(1M) -(EcoRI) | Forward (5’-3’) | CCGGAATTCGCCACCATGGATCCCGGGCA GCAGC |
| YAP(154T) -(SalI) | Reverse (5’-3’) | GGACCGTCGACTGTAGCTGCTGGGCCAGA GACTACTCC |
| YAP(155P) -(EcoRI) | Forward (5’-3’) | CCGGAATTCGCCACCATGGATCCCACAGC TCAGCATCTTCGACA |
| YAP(504L) -(SalI) | Reverse (5’-3’) | GGACCGTCGACTAACCATGTAAGAAAGCT TTCTTTATCTAGCTTGG |
| YAP(263L) -(SalI) | Reverse (5’-3’) | GGACCGTCGACAAGCCTTGGGTCTAGCCA AGAGG |
| YAP(264D) -(EcoRI) | Forward (5’-3’) | CCGGAATTCGCCACCATGGACCCTCGTTTT GCCATGAACC |
| YAP(287P) -(SalI) | Reverse (5’-3’) | GGACCGTCGACGGGAGCCAGGGGTGGTG G |
| YAP(288Q) -(EcoRI) | Forward (5’-3’) | CCGGAATTCGCCACCATGGATCAGAGCCC ACAGGGAGGCG |
| YAP(499S) -(SalI) | Reverse (5’-3’) | GGACCGTCGACGCTTTCTTTATCTAGCTTG GTGGCAGCC |

**Supplementary Table 13. Sequences of siRNA Used in This Study**

| | | |
|---|---|---|
| lncARSR siRNA | Sense (5'-3') | CAACCCUGGAUCCAAAGUATT |
| | Antisense (5'-3') | UACUUUGGAUCCAGGGUUGTT |
| YAP siRNA-1 | Sense (5'-3') | GGUGAUACUAUCAACCAAATT |
| | Antisense (5'-3') | UUUGGUUGAUAGUAUCACCTT |
| YAP siRNA-2 | Sense (5'-3') | CUGCCACCAAGCUAGAUAATT |
| | Antisense (5'-3') | UUAUCUAGCUUGGUGGCAGTT |
| TEAD1 siRNA | Sense (5'-3') | CUGCCAUUCAUAACAAGCUTT |
| | Antisense (5'-3') | AGCUUGUUAUGAAUGGCAGTT |
| TEAD2 siRNA | Sense (5'-3') | GCCAGAUGCAGUUGAUUCUTT |
| | Antisense (5'-3') | AGAAUCAACUGCAUCUGGCTT |
| TEAD3 siRNA | Sense (5'-3') | CCAGUGUCCUGCAGAACAATT |
| | Antisense (5'-3') | UUGUUCUGCAGGACACUGGTT |
| TEAD4 siRNA | Sense (5'-3') | GAACGUCCCAUGAUGUGAATT |
| | Antisense (5'-3') | UUCACAUCAUGGGACGUUCTT |
| TEAD1/3/4 siRNA | Sense (5'-3') | UGAUCAACUUCAUCCACAATT |
| | Antisense (5'-3') | UUGUGGAUGAAGUUGAUCATT |
| LATS1 siRNA | Sense (5'-3') | GCAGCGUCUACAUCGUAAATT |
| | Antisense (5'-3') | UUUACGAUGUAGACGCUGCTT |
| LATS2 siRNA | Sense (5'-3') | CUACCAGAAAGAGUCUAAUTT |
| | Antisense (5'-3') | AUUAGACUCUUUCUGGUAGTT |
| Dicer siRNA | Sense (5'-3') | GGGCACCCAUCUCUAAUUATT |
| | Antisense (5'-3') | UAAUUAGAGAUGGGUGCCCTT |
| ANXA6 siRNA-1 | Sense (5'-3') | GGGACUUUGAGAAGCUAAUTT |
| | Antisense (5'-3') | AUUAGCUUCUCAAAGUCCCTT |
| ANXA6 siRNA-2 | Sense (5'-3') | CUCGGACCAAUGCUGAAAUTT |
| | Antisense (5'-3') | AUUUCAGCAUUGGUCCGAGTT |
| FARSB siRNA-1 | Sense (5'-3') | CACCUACACUGACGAAGAATT |
| | Antisense (5'-3') | UUCUUCGUCAGUGUAGGUGTT |
| FARSB siRNA-2 | Sense (5'-3') | CCUGUAUCCAGUUAUCUAUTT |
| | Antisense (5'-3') | AUAGAUAACUGGAUACAGGTT |
| HSPA8 siRNA-1 | Sense (5'-3') | GUCCUCAUCAAGCGUAAUATT |
| | Antisense (5'-3') | UAUUACGCUUGAUGAGGACTT |
| HSPA8 siRNA-2 | Sense (5'-3') | GCUGGUCUCAAUGUACUUATT |
| | Antisense (5'-3') | UAAGUACAUUGAGACCAGCTT |
| IGF2BP1 siRNA-1 | Sense (5'-3') | GGCCCAUAAUAACUUUGUATT |
| | Antisense (5'-3') | UACAAAGUUAUUAUGGGCCTT |
| IGF2BP1 siRNA-2 | Sense (5'-3') | GCUCCCUAUAGCUCCUUUATT |
| | Antisense (5'-3') | UAAAGGAGCUAUAGGGAGCTT |
| LTA4H siRNA-1 | Sense (5'-3') | CAAAGGACCUUACAAUAGATT |
| | Antisense (5'-3') | UCUAUUGUAAGGUCCUUUGTT |
| LTA4H siRNA-2 | Sense (5'-3') | CAUGAAAUAUCUCAUAGCUTT |
| | Antisense (5'-3') | AGCUAUGAGAUAUUUCAUGTT |
| PRMT5 siRNA-1 | Sense (5'-3') | CCGGACUUUGUGUGACUAUTT |
| | Antisense (5'-3') | AUAGUCACACAAAGUCCGGTT |
| PRMT5 siRNA-2 | Sense (5'-3') | GGUGAACACAGUACUACAUTT |
| | Antisense (5'-3') | AUGUAGUACUGUGUUCACCTT |
| SRP68 siRNA-1 | Sense (5'-3') | GAGCUUCUGACCGAUAAUATT |
| | Antisense (5'-3') | UAUUAUCGGUCAGAAGCUCTT |
| SRP68 siRNA-2 | Sense (5'-3') | CAGCUACCAUGAGUGAAGUTT |
| | Antisense (5'-3') | ACUUCACUCAUGGUAGCUGTT |
| ZNF185 siRNA-1 | Sense (5'-3') | CCACUGAGGAUUACAAGAATT |
| | Antisense (5'-3') | UUCUUGUAAUCCUCAGUGGTT |
| ZNF185 siRNA-2 | Sense (5'-3') | CAACUUGUCAGACGAGAGATT |
| | Antisense (5'-3') | UCUCUCGUCUGACAAGUUGTT |
| Ctrl siRNA | Sense (5'-3') | UUCUCCGAACGUGUCACGUTT |
| | Antisense (5'-3') | ACGUGACACGUUCGGAGAATT |

**Supplementary Table 14. Sequences of Primers Used for qRT-PCR in This Study**

| | | |
|---|---|---|
| lncARSR | Forward(5'-3') | TTTGAAATGCTCTTTGAGGGAT |
| | Reverse(5'-3') | TGCAGGTTGTCTGAAGTTGGA |
| Oct4 | Forward(5'-3') | CTTGCTGCAGAAGTGGGTGGAGGAA |
| | Reverse(5'-3') | CTGCAGTGTGGGTTTCGGGCA |
| Sox2 | Forward(5'-3') | AAATGGGAGGGGTGCAAAAGAGGAG |
| | Reverse(5'-3') | CAGCTGTCATTTGCTGTGGGTGATG |
| Nanog | Forward(5'-3') | AATACCTCAGCCTCCAGCAGATG |
| | Reverse(5'-3') | TGCGTCACACCATTGCTATTCTTC |
| Klf4 | Forward(5'-3') | GCCCCTCGGGCGGCTTCGTGGCCGAGCTC |
| | Reverse(5'-3') | CGTACTCGCTGCCAGGGGCG |
| c-Myc | Forward(5'-3') | CATCATCATCCAGGACTGTATGTG |
| | Reverse(5'-3') | GGCTGCCGCTGTCTTTGC |
| CD105 | Forward(5'-3') | CACTAGCCAGGTCTCGAAGG |
| | Reverse(5'-3') | CTGAGGACCAGAAGCACCTC |
| CD133 | Forward(5'-3') | GCAGCAGTCTGACCAGCGTGAA |
| | Reverse(5'-3') | ACGGGTGGAAGCTGCCTCAGTT |
| YAP | Forward(5'-3') | AGCCCAAATCCCACTCCC |
| | Reverse(5'-3') | GTATCTCAAAAGAAGACTGTCGAAGA |
| CTGF | Forward(5'-3') | GCCCAAGGACCAAACCG |
| | Reverse(5'-3') | GTGCAGCCAGAAAGCTCAAA |
| INHBA | Forward(5'-3') | TCGGGGAGAACGGGTATG |
| | Reverse(5'-3') | TGCTGGAGACAGGGAAGACA |
| BIRC5 | Forward(5'-3') | TTCTCAAGGACCACCGCATCT |
| | Reverse(5'-3') | CGCACTTTCTCCGCAGTTTC |
| CYR61 | Forward(5'-3') | AGTGCTGCGAGGAGTGGG |
| | Reverse(5'-3') | GGTTGTATAGGATGCGAGGCT |
| ANKRD1 | Forward(5'-3') | GAGGAACTGGTCACTGGAAAGA |
| | Reverse(5'-3') | GGGTCACAGGGTGGGCTA |
| TEAD1 | Forward(5'-3') | CATCTTATCAGACGAAGGCAAAA |
| | Reverse(5'-3') | AGGTCGGGCGTGGAATC |
| TEAD2 | Forward(5'-3') | GTCTGATGAAGGCAAGATGTATGG |
| | Reverse(5'-3') | CAAGGTGAACGGTGTCTGTGAG |
| TEAD3 | Forward(5'-3') | AGGACGGGGAAGACTCGG |
| | Reverse(5'-3') | TGGGGTAGGCTGGCTGTG |
| TEAD4 | Forward(5'-3') | GGGGACCCTCCAATGCCT |
| | Reverse(5'-3') | CTCTGTCTCAACTTTCTCCACCA |
| β-actin | Forward(5'-3') | AATCGTGCGTGACATTAAGGAG |
| | Reverse(5'-3') | ACTGTGTTGGCGTACAGGTCTT |

**Supplementary Table 15. Primary Antibodies Used in This Study**

| Antigens | Manufacturer | Application |
|---|---|---|
| Sox2 | Abcam | 1:1000 for WB |
| Oct4 | Abcam | 1:1000 for WB |
| p-YAP $^{S127}$ | Cell Signaling Technology | 1:1000 for WB |
| p-YAP $^{S381}$ | Cell Signaling Technology | 1:1000 for WB |
| YAP | Cell Signaling Technology | 1:50 for IP, 1:100 for ICC, 1:1000 for WB,1:100 for IHC, 1:50 for RIP, 1:50 for ChIP |
| LATS1 | Cell Signaling Technology | 1:50 for IP, 1:1000 for WB |
| LATS2 | Proteintech | 1:1000 for WB |
| TEAD1 | Cell Signaling Technology | 1:50 for ChIP |
| FLAG | Merck Millipore | 1:50 for RIP, 1:1000 for WB, 1:100 for ICC, 1:50 for ChIP |
| MYC | Cell Signaling Technology | 1:50 for IP |
| CD105-APC | ebioscience | 1:50 for FACS |
| CD133-FITC | ebioscience | 1:50 for FACS |
| Histone H3 | Abcam | 1:1000 for WB |
| HSPA8 | Proteintech | 1:1000 for WB |
| IGF2BP1 | Proteintech | 1:1000 for WB |
| LTA4H | Proteintech | 1:1000 for WB |
| GAPDH | Abclonal | 1:5000 for WB |

WB, western blot; IHC, immunohistochemitry; ICC, Immunocytochemistry;
IP, immunoprecipitation; ChIP, chromatin immunoprecipitation;
RIP, RNA immunoprecipitation, FACS, fluorescence activated cell sorter.

**Supplementary Table 16. Sequences of Primers Used for ChIP-qPCR in This Study**

| | | |
|---|---|---|
| TB 1 | Forward(5’-3’) | TCACTGGGTGTCTGGTCTGC |
| | Reverse(5’-3’) | GAGCGTTGCCTTGGGAG |
| TB 2 | Forward(5’-3’) | GGAAGAAAGCAGCCAACAACC |
| | Reverse(5’-3’) | GACGCCTACCGCAACG |
| Neg | Forward(5’-3’) | ATCTTACTTCGTTCGGTTCACATC |
| | Reverse(5’-3’) | GCGACTCCATTTCTGGCTCTA |
